# Anti-Neuroinflammatory Agent, Restricticin B, from the Marine-Derived Fungus *Penicillium janthinellum* and Its Inhibitory Activity on the NO Production in BV-2 Microglia Cells

**DOI:** 10.3390/md18090465

**Published:** 2020-09-14

**Authors:** Byeoung-Kyu Choi, Song-Hee Jo, Dong-Kug Choi, Phan Thi Hoai Trinh, Hwa-Sun Lee, Cao Van Anh, Tran Thi Thanh Van, Hee Jae Shin

**Affiliations:** 1 Marine Natural Products Chemistry Laboratory, Korea Institute of Ocean Science and Technology, 385 Haeyang-ro, Yeongdo-gu, Busan 49111, Korea; choibk4404@kiost.ac.kr (B.-K.C.); hwasunlee@kiost.ac.kr (H.-S.L.); caovananh@kiost.ac.kr (C.V.A.); 2 Department of Applied Life Science, Graduate school of Konkuk University, Chungju 27478, Korea; wowsong333@naver.com (S.-H.J.); choidk@kku.ac.kr (D.-K.C.); 3 Department of Marine Biotechnology, Nhatrang Institute of Technology Research and Application, Vietnam Academy of Science and Technology, 02 Hung Vuong, Nha Trang 650000, Vietnam; phanhoaitrinh84@gmail.com (P.T.H.T.); tranthanhvan@nitra.vast.vn (T.T.T.V.); 4 Department of Marine Biotechnology, University of Science and Technology (UST), 217 Gajungro, Yuseong-gu, Daejeon 34113, Korea

**Keywords:** restricticin, marine-derived fungus, *Penicillium janthinellum*, BV-2 microglia cells, anti-neuroinflammatory

## Abstract

A new compound containing a triene, a tetrahydropyran ring and glycine ester functionalities, restricticin B (**1**), together with four known compounds (**2**–**5**) were obtained from the EtOAc extract of the marine-derived fungus *Penicillium janthinellum*. The planar structure of **1** was determined by detailed analyses of MS, 1D and 2D NMR data. The relative and absolute configurations of **1** were established via the analyses of NOESY spectroscopy data, the comparison of optical rotation values with those of reported restricticin derivatives and electronic circular dichroism (ECD). All the compounds were screened for their anti-neuroinflammatory effects in lipopolysaccharide (LPS)-induced BV-2 microglia cells. Restricticin B (**1**) and *N*-acetyl restricticin (**2**) exhibited anti-neuroinflammatory effects by suppressing the production of pro-inflammatory mediators in activated microglial cells.

## 1. Introduction

The novel class of potent antifungal agents, restricticin and restricticinol, were first isolated from the fermentation broth of *Penicillium restrictum* [1,2]. These compounds contain a tetrahydropyran ring and triene side chains in common and restricticin possesses a glycine unit linked to the tetrahydropyran ring [3]. The initial biological activity study of restricticin derivatives displayed that only restricticin exhibited potent antifungal activity against both yeast and filamentous fungi [1,4]. The very closely related compounds with a shorter polyene chain, lanomycin and lanomycinol, were isolated from *Pycnidiophora dispersa* in 1992 and chaunopyran A possessing a same polyene chain and 2-alkenyl-tetrahydropyran was reported in 2017 [5,6]. Lanomycin was active against some *Candida* species and dermatophytes and not active against Gram-positive, Gram-negative bacteria and *Aspergillus fumigatus* [7]. Restricticin and lanomycin were first reported as natural antifungal agents to inhibit cytochrome P_450_ lanosterol demethylase [5,8]. Interestingly, restricticinol and lanomycinol did not show such antifungal activity [8]. Because of the considerable interest in their biological activity, several methods for the total synthesis of the antifungal agents have been described [8,9,10].

Microglia, macrophages and representatives of the innate immune system in the brain, have been implicated as active contributors to neuron damage and readily become activated in response to infection or injury [11,12]. Activated microglia upregulate pro-inflammatory and neurotoxic mediators including tumor necrosis factor-*α* (TNF-*α*), interleukin-1*β* (IL-1*β*), interleukin-6 (IL-6) and free radicals such as nitric oxide (NO) and superoxide [13]. Moreover, large numbers of activated microglia are present in the CNS tissue of patients with chronic neurodegenerative diseases such as Parkinson’s disease, Alzheimer’s disease, Huntington’s disease and prion disease [14,15]. Therefore, the suppression of pro-inflammatory and neurotoxic molecules in activated microglia would be an effective therapeutic approach to treat various neuronal diseases [16].

**Figure 1:**
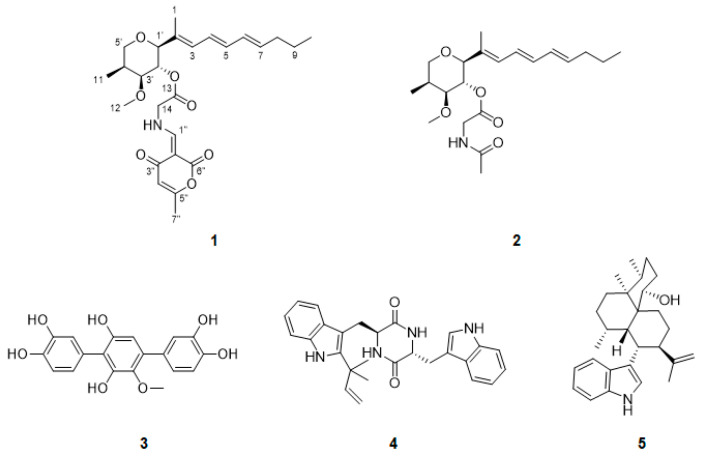
Structures of compounds **1**–**5** isolated from *Penicillium janthinellum.*

Over the past decade, the secondary metabolites from marine microbes have been researched as a promising source for medical applications and clinical development [17,18]. Among the marine-derived microorganisms, marine fungi produce bioactive compounds with a broad range of bioactivities such as antibacterial, antiviral, antitumoral, antimalarial and anti-inflammatory [19]. As a part of our ongoing investigation for diverse secondary metabolites from marine microorganisms, we isolated the fungal 168CLC-17.1 strain from a sediment sample collected from Cu Lao Cham Island, Vietnam, and based on its nuclear ribosomal internal transcribed spacer (ITS) region sequence, it was identified as *Penicillium janthinellum*. Further chemical investigation of the ethyl acetate extracts of the fungal culture broth yielded a new restricticin derivative, named restricticin B (**1**), together with four known compounds, *N*-acetyl restricticin (**2**) [19], 3,3″-dihydroxy-6′-desmethyl terphenyllin (**3**) [20], fellutanine B (**4**) [21] and 10,23-dihydro-24,25-dehydro aflavinin (**5**) [22] (Figure 1). Here, we describe the chemical investigation, structural determination, and bioactivities of these compounds.

## 2. Results and Discussion

**Figure 2:**
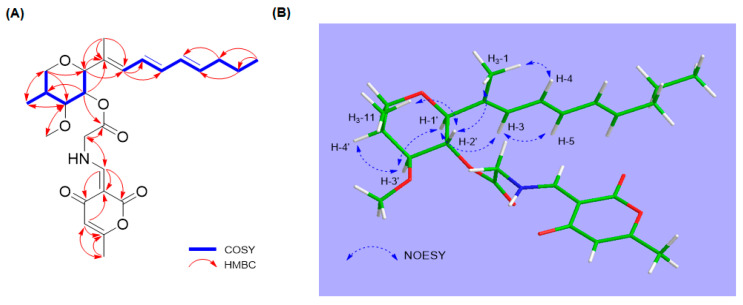
(**A**) Key COSY and HMBC correlations of **1**. (**B**) Key NOESY correlations of **1**.

Compound **1** was isolated as a pale brown oil and its molecular formula was determined to be C_26_H_35_NO_7_ based on the HRESIMS ion at *m*/*z* 496.2306 ([M + Na]^+^ calcd. 496.2311). The ^1^H NMR spectrum of **1** indicated characteristic signals of seven olefinic protons (*δ*_H_ 8.20, 6.27, 6.21, 6.08, 5.98, 5.74, and 5.70), four methine protons (*δ*_H_ 5.00, 3.60, 3.52, and 2.33), four methylene protons (*δ*_H_ 4.32, *δ*_H_ 3.78, 3.63, *δ*_H_ 2.05, and *δ*_H_ 1.40) and five methyl protons (*δ*_H_ 3.33, 2.51, 1.74, 1.06, and 0.90). The ^13^C NMR spectrum, in combination with the HSQC spectrum, displayed the presence of 26 carbon signals, three carbonyl carbons (*δ*_C_ 184.1, 164.9, and 167.2), five pairs of olefinic carbons (*δ*_C_ 165.4, 162.7, 135.3, 134.4, 132.5, 130.6, 129.5, 125.3, 106.9, and 96.6), three oxygenated methine carbons (*δ*_C_ 84.9, 81.0, and 70.4), one methine carbon (*δ*_C_ 32.1), one oxygenated and one nitrogenated methylene carbons (*δ*_C_ 70.2 and 50.0, respectively), two methylene carbons (*δ*_C_ 34.5 and 22.0), one methoxy carbon (*δ*_C_ 55.0), and four methyl carbons (*δ*_C_ 18.7, 12.6, 10.4, and 9.5). The planar structure of **1** was elucidated by the analysis of the 2D NMR data, including the COSY and HMBC spectra (Figure 2). The COSY correlations of H-3/H-4/H-5/H-6/H-7/H_2_-8/H_2_-9/H_3_-10 revealed the presence of an unsaturated side chain. The presence of the tetrahydropyran ring was confirmed by the COSY correlations of H-1′/H-2′/H-3′/H-4′/H_2_-5′/H_3_-11 and the HMBC correlations from H-5′ to C-1′, H_3_-12 to C-3′, and H_3_-11 to C-3′ and C-5′. The HMBC correlations from H_3_-1 to C-1′, C-2, and C-3, H-1′ to C-2 and C-3, and H-2′ to C-2 indicated that the triene side chain is connected to the tetrahydropyran ring. The geometry of the side chain was determined as all *E* by the large ^3^*J*_H,H_ coupling constants for H-4/H-5 (14.5 ppm) and H-6/H-7 (15.0 ppm) and the NOESY correlations of H-3/H-5 and H_3_-1/H-4. Afterwards, a glycine moiety was confirmed by the HMBC correlations from H-2′ and H_2_-14 to C-13. Detailed analysis of 1D and 2D NMR spectra of **1** revealed that the partial structure was closely similar to that of restricticin. The HMBC correlations from H-1″ to C-2″, C-3″, C-6″, and C-14, H-4″ to C-2″ and C-5″, and H-7″ to C-4″ and C-5″ established the α-pyrone moiety linked to the NH of the glycine unit.

**Scheme 1:**
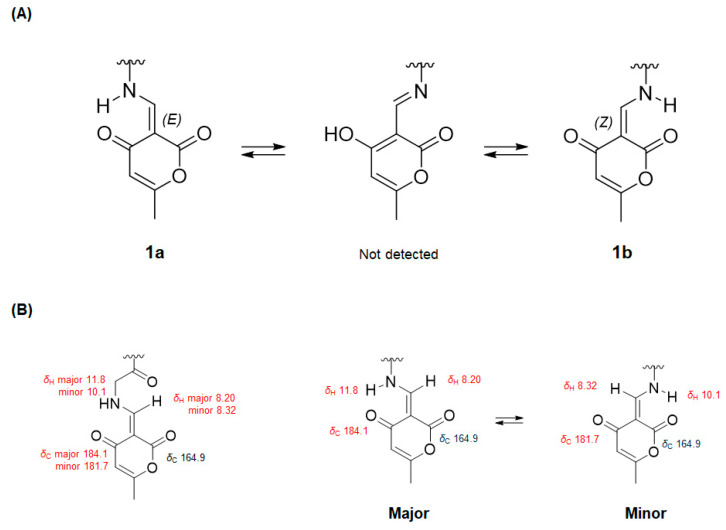
(**A**) Tautomeric equilibrium of compound **1**. (**B**) Comparison of ^1^H and ^13^C chemical shifts of **1a** (major) and **1b** (minor) based on the interconversion of keto-enamine forms.

Interestingly, the NMR data in one specific area showed a duplication of the proton and carbon signals (Table 1). The ^1^H NMR spectrum of **1** displayed a pair of olefinic singlet signals at H-1″ (major: *δ*_H_ 8.20, minor: *δ*_H_ 8.32) and doublet methylene signals at H-14 (major: *δ*_H_ 4.27, 4.36, minor: *δ*_H_ 4.29, 4.38). Moreover, a pair of signals for the exchangeable NH proton (major: *δ*_H_ 11.8, minor: *δ*_H_ 10.1) was detected in the ^1^H NMR spectrum in CDCl_3_. The ^13^C NMR spectrum also showed that two sets of the carbon signals at C-13 (*δ*_C_ 167.1 and 167.2), C-14 (*δ*_C_ 49.9 and 50.0), C-1″ (*δ*_C_ 161.5 and 162.7), C-2″ (*δ*_C_ 96.5 and 96.6), C-3″ (*δ*_C_ 181.7 and 184.1), C-4″ (*δ*_C_ 106.6 and 106.9) and C-5″ (*δ*_C_ 165.3 and 165.4). These phenomena were presumed to be involved in imine–enamine tautomerism involving the ketone group at C-3″. The form of **1a** was probably explained as the *E* geometry of Δ^1″^, based on NMR evidence via tautomeric equilibrium, where an exchangeable amide proton is notably shifted downfield by a strong intramolecular hydrogen bond with a ketocarbonyl group (C-3″) (Scheme 1). Otherwise, the proton signal of **1b**-NH in the more upfield region was assigned as the *Z* geometry of Δ^1″^ due to the weaker hydrogen bond with a carbonyl group (C-6″) than **1a**. For the same reason, the H-1″ of **1a** and **1b** also displayed the similar pattern.

**Scheme 2:**
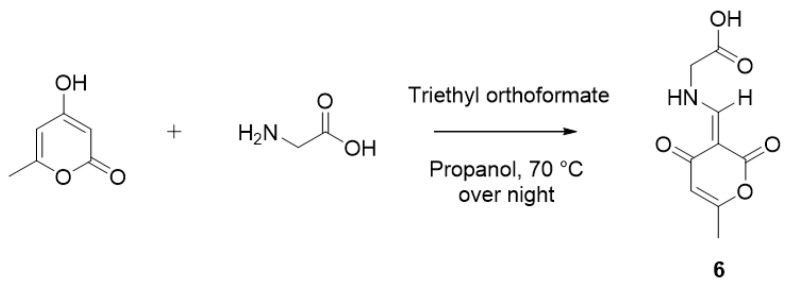
Synthesis of the pyrone moiety (**6**) of restricticin B.

To rationalize these unexpected observations, EXSY (exchange spectroscopy) experiments and the synthesis of the pyrone moiety were carried out following previous studies [23,24]. In the NOESY spectrum, EXSY correlations (equilibrium spots) of NH and H-1″ confirmed the presence of two isomers as an inseparable mixture (Figure S9, Supporting Information). An extensive literature survey has indicated that 4-hydroxy-6-methyl-2-pyrone and glycine in 2-propanol in the presence of triethyl orthoformate led to **6** (Scheme 2), and the ^1^H NMR spectra in previous studies displayed a pair of signals due to the existence of *E* and *Z* isomers [24,25]. Comparison of the NMR data of **1** and **6** indicated that they had the same phenomenon of imine–enamine tautomerism (Figure S10, Supporting Information). Thus, the planar structure of **1** was assigned as shown in Figure 1.

**Figure 3:**
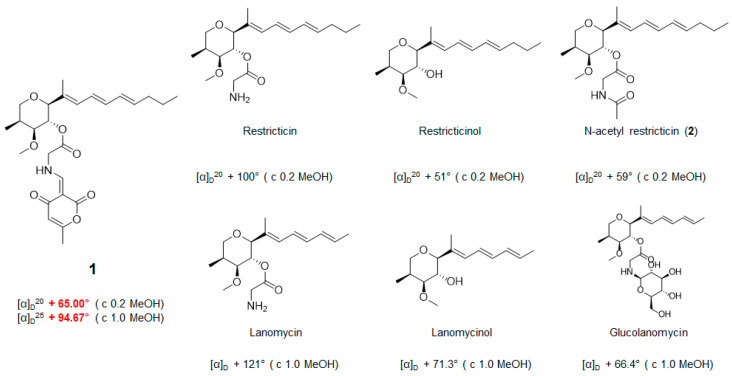
Comparison of the optical rotation value of **1** with reference compounds.

**Figure 4:**
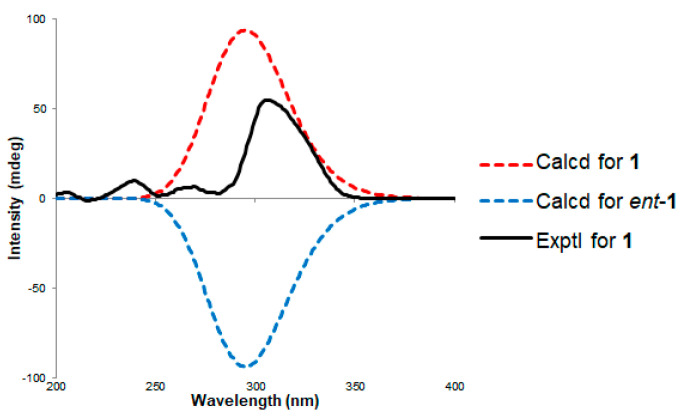
Comparison of the circular dichroism (CD) curves between the experimental and calculated data of **1**.

The relative stereochemistry of **1** was determined by the analysis of NOESY spectra and the comparison of the optical rotation values with the literature. The strong NOESY correlations of H_3_-1/H-2′/H_3_-11 and H-1′/H-3′/H-4′ suggested that H_3_-1, H-10 and H_3_-11 were on the same face, and H-1′, H-3′ and H-4′ were on the opposite face. Finally, the absolute configuration of **1** was confirmed by the comparison of the specific rotation value of **1** with those in the literature [8,9,10,26] (Figure 3) and the electronic circular dichroism (ECD) method (Figure 4). The optical rotation value of **1** is in good agreement with all reported restricticin, lanomycins and *N*-acetyl restricticin (**2**), suggesting that **1** has the same absolute configuration with its derivatives as shown in Figure 3. Additionally, the ECD calculation of enantiomers (**1**: 1′*S*,2′*R*,3′*S*,4′*S*, *ent*-**1**: 1′*R*,2′*S*,3′*R*,4′*R*) was carried out at B3LYP/6-311 + G(d)1 level. The experimental CD spectrum showed a positive Cotton effect at 305 nm. Then, the theoretical ECD spectrum of **1a** was in reasonable agreement with the measured CD spectrum, suggesting that the absolute configurations of **1** are defined as *1*′S,*2*′R,*3*′S,*4*′S. Based on these results, the structure of **1** was assigned and named restricticin B.

The structures of the four known compounds were determined as *N*-acetyl restricticin (**2**), 3,3″-dihydroxy-6′-desmethyl terphenyllin (**3**), fellutanine B (**4**) and 10,23-dihydro-24,25-dehydro aflavinin (**5**) by comparing their ^1^H, ^13^C NMR and MS data with those reported in the literature (Supporting Information).

Compounds **1** and **2** were tested for their antimicrobial activity using fungi (*Penicillium italicum* KCTC 6437, *Rhizopus oryzae* KCTC 6944), yeast (*Candida albicans* KCTC 7678), Gram-positive bacteria (*Micrococcus lutes* KCTC1915, *Staphylococcus auresus* KCTC 1927, *Bacillus subtilis* KCTC 1021) and Gram-negative bacteria (*Salmonella typhimurium* KCTC 2515, *Klebsiella pneumoniae* KCTC 2690, *Escherichia coli* KCTC 2441) and the cytotoxicity against cancer cell lines (HCT-15, NUGC-3, NCI-H23, ACHN, PC-3 and MDA-MB-231). However, **1** and **2** showed no antibacterial activity and cytotoxicity against cancer cell lines. Along with the fact that restricticinol and lanomycinol have no antifungal activity in previous research, the results of this study suggested that the free amine of the glycine unit is important for the antifungal activity.

**Figure 5:**
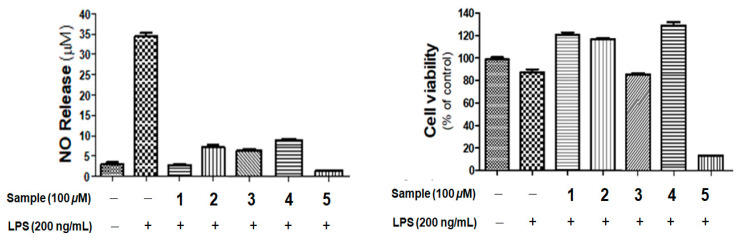
The effects of compounds **1**–**5** on NO production in lipopolysaccharide (LPS)-stimulated BV-2 cells. Cells were pretreated with the indicated concentration of samples for 1 h and then stimulated with LPS (100 ng/mL) for 24 h. Cell viability was evaluated using MTT assay. Results are shown as percentage of control samples. Values are the mean ± standard error.

All the isolated compounds (**1**–**5**) were evaluated for inhibitory activity on nitric oxide (NO) production in lipopolysaccharide (LPS)-stimulated BV-2 microglial cells and for their cytotoxicity. The cells were initially treated with 100 μΜ of each compounds and/or LPS (200 ng/mL) to measure the levels of NO and cytotoxicity using an MTT assay. As shown in Figure 5, all the compounds inhibited the LPS-induced NO production in BV-2 microglial cells. However, compounds **3** and **5** displayed weak and strong toxicity, respectively. Compounds **1**, **2**, and **4** decreased the production of NO without showing cytotoxicity at the treated concentrations. According to the results, restricticin derivatives have the most potent anti-inflammatory effect among the treated compounds without cytotoxicity. Therefore, restricticin B (**1**) and *N*-acetyl restricticin (**2**) were selected to further investigate the effects on the LPS-induced expression of inducible nitric oxide synthase (iNOS) and cyclooxygenase-2 (COX-2) mRNA and protein expression, and the production of LPS-stimulated pro-inflammatory cytokines in BV-2 microglia cells.

**Figure 6:**
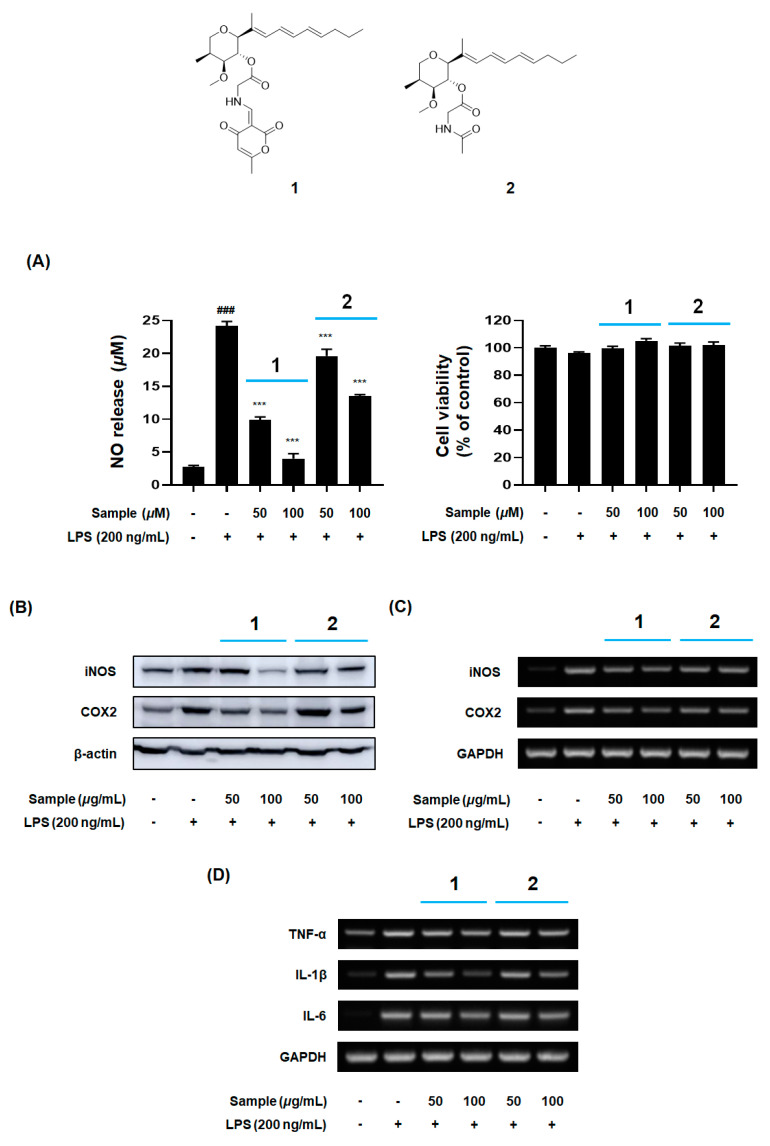
(**A**) The measurements of nitrite levels in the culture media were performed using the Griess reaction. Cell viability was evaluated using the MTT assay. Results are shown as the percentage of control samples. (**B**) Inhibition of iNOS and COX-2 protein (**C**) and mRNA (**D**) and pro-inflammatory cytokines expression by compounds **1**–**2** in LPS-stimulated BV-2 cells. The data (**B**–**D**) are expressed as the relative signal intensity for two independent experiments. Values are the mean ± standard error. ### *p* < 0.001, vs. control group and *** *p* < 0.001 vs. LPS-treated group.

Two restricticin class compounds (**1** and **2**) at the concentrations of 50 μM and 100 μM inhibited the NO production in BV-2 cells in a dose-dependent manner (Figure 6A). Compound **1** showed a stronger inhibition of NO production than **2**. As shown in Figure 6B,C, LPS treatment (200 ng/mL) significantly upregulated iNOS and COX-2 expression, and the mRNA and protein expression levels of iNOS and COX-2 were inhibited by restricticins in concentration-dependent manner. To investigate whether restricticins repress the production of pro-inflammatory cytokines such as TNF-*α*, IL-1*β* and IL-6, RT-PCR was performed, and the results showed that restricticins reduced the secretion of pro-inflammatory cytokines. Interestingly, IL-1*β* was the most strongly inhibited by compound **1** at 100 μM. Consequently, these results indicated that restricticins have effective properties in neuroinflammation processes and suppress the expression of pro-inflammatory mediators at the transcriptional level.

## 3. Materials and Methods

### 3.1. General Experimental Procedures

NMR spectra (^1^H, ^13^C, COSY, HSQC, HMBC and NOESY) were measured on a Varian Unity 500 MHz (Varian Inc., Palo Alto, CA, USA) and a Bruker 600 MHz (Bruker BioSpin GmbH, Rheinstetten, Germany) spectrometer with TMS as an internal standard. Optical rotations were obtained on a Rudolph Research Analytical Autopol III polarimeter (Rudolph Research Analytical, Hackettstown, NJ, USA). CD spectra were recorded on a JASCO J-1500 spectrometer (JASCO Corporation, Tokyo, Japan). UV spectra were acquired on a Shimadzu UV-1650PC spectrophotometer (Shimadzu Corporation, Kyoto, Japan). IR spectra were measured on a JASCO FT/IR-4100 spectrophotometer (JASCO Corporation, Tokyo, Japan). HRESIMS were measured on Waters Synapt HDMS LC/MS mass spectrometer (Waters Corporation, Milford, MA, USA). HPLC system was composed of a PrimeLine Binary pump (Analytical Scientific Instruments, Inc., El Sobrante, CA, USA) with RI-101 (Shoko Scientific Co. Ltd., Yokohama, Japan), semi-preparative ODS column (YMC-Pack-ODS-A, 250 × 10 mm i.d, 5 µm) and an analytical ODS column (YMC-Pack-ODS-A, 250 × 4.6 mm i.d, 5 µm)(YMC Corporation, Kyoto, Japan).

### 3.2. BV-2 Microglial Cell Culture and Treatment

The BV-2 murine microglial cells were cultured in DMEM (Dulbecco’s modified Eagle medium) supplemented with 5% fetal bovine serum and 1% penicillin/streptomycin (100 units/mL) at 37 °C in a humidified 5% CO_2_ incubator. The cell was seeded at a density of 2.5 × 10^5^ cells/mL and pretreated with the same concentration of samples and followed by LPS incubation (200 ng/mL) [27].

### 3.3. Cell Viability and Nitrite Assay

The BV-2 microglial cells seeded at a density of 2.5 × 10^5^ cells/well were pretreated with 50~100 μM of compounds **1**–**5** for 1 h, followed by LPS (200 ng/mL) for 24 h. Twenty microliters of MTT solution (2 mg/mL, conc.) was added to each well in 24 wells. After 1 h, the supernatant was sucked and dissolved the formazan crystals in viable cells from DMSO. Optical density was measured at 550 nm using a microplate reader and values were determined in comparison to control cells. For the nitrite assay, the BV-2 microglial cells seeded at a density of 2.5 × 10^5^ cells/well were pretreated with 50~100 μM of compounds **1**–**5** for 1 h, followed by LPS (200 ng/mL) for 24 h. One hundred microliters of supernatants was collected and transferred to new microplate. After transfer, the supernatants were mixed with an equal volume of Griess reagent for 10 min at room temperature in the dark. The amount of nitrite in each sample was measured by using a range of sodium nitrite dilutions as standard solutions. Absorbance was determined at 540 nm using a microplate reader [27].

### 3.4. Total RNA Extraction and Reverse Transcription Polymerase Chain Reaction (RT-PCR)

Total RNA extraction of BV-2 microglial cells was prepared using Trizol reagent following the manufacturer’s instructions. RNA (2.5 μg) was reverse-transcribed using ReverTra Ace-*α* kit and PCR amplification was carried out using specific primers [28].

### 3.5. Western Blot Analysis

Treated BV-2 cells (5 × 10^5^ cells/well) were washed twice with PBS and lysed for 10 min using RIPA lysis buffer at 4 °C to obtain the total cell lysate. The cells were centrifuged at 14,000 rpm at 4 °C and collected and separately stored for further analysis. DC Protein Assay kit was used for the protein concentration of isolated compounds. Equal amounts of protein (35 µg for cells) were separated by 10% sodium dodecyl sulfate–polyacrylamide electrophoresis, and the resolved proteins were transferred to polyvinylidene difluoride membranes. The membranes were incubated for 1 h with 5% skim-milk in TBS buffer to block nonspecific binding. The blots were visualized by a PowerOpti-ECL kit obtained from the detection system (Animal Genetics Inc., Tallahassee, FL, USA) [28].

### 3.6. Fungal Strain and Fermentation

The strain 168CLC-17.1 was isolated from a sediment sample, and collected at Cu Lao Cham Island, Quang Nam, Vietnam in August 2016. The strain was identified by DNA amplification and the sequencing of the ITS region (GenBank accession number AB293968.1) and named as *Penicillium janthinellum* 168CLC-17.1. The cultivation and fermentation of the fungal strain have been previously described [29].

### 3.7. Isolation of Compounds **1**–**5**

The mycelia and rice media were extracted with EtOAc and then concentrated to yield a crude extract (6 g). The crude extract was partitioned on ODS by flash column chromatography on using a gradient of MeOH/H_2_O (1:4, 2:3, 3:2, 4:1 and 100% MeOH, each fraction 300 × 3). The third fraction eluted with 80% MeOH was purified with a semi-preparative reversed-phase HPLC (2.0 mL/min, RI detector, 22% MeCN/H_2_O) to obtain **1** (7.5 mg, *t*_R_ = 28 min) and **2** (3.2 mg, *t*_R_ = 18 min). The first fraction eluted with 40% MeOH was further fractionated on ODS by column chromatography, eluting with a step gradient of MeOH/H_2_O (20:80 to 40:60, *v*/*v*) to give ten fractions (Fr. A-H). Fr. B (82 mg) was subjected to ODS HPLC (YMC-Pack-ODS-A, 250 × 10 mm i.d, 5 µm, flow rate 3.0 mL/min, RI detector) with 25% MeOH in H_2_O to yield compound **3** (62 mg, *t_R_* = 14 min). Compound **4** (5.1 mg, *t*_R_ = 46 min) was purified from the third fraction eluted with 60% MeOH by a semi-preparative reversed-phase HPLC (3.0 mL/min, RI detector, 55% MeOH/H_2_O). The first fraction eluted with 100% MeOH was further partitioned by column chromatography on ODS eluting with a step gradient of MeOH/H_2_O (90:10 to 100:0, *v*/*v*) to yield ten fractions (Fr. A-J). Fr. C-F (182 mg) was purified by a ODS HPLC (YMC-Pack-ODS-A, 250 × 10 mm i.d, 5 µm, flow rate 4.0 mL/min, RI detector) with 95% MeOH in H_2_O to yield 5 (85 mg, *t*_R_ = 12 min).

Restricticin B (**1**): pale brown oil; ${\left[\alpha \right]}_{D}^{20}$ + 65 (c 0.2, MeOH); CD (MeOH), λ_max_ (Δ*ε*) 305 (54.59) nm; IR ν_max_ 2933, 1727, 1653, 1617, 1458, 1328, 1201, 1109, 1035, 985 cm^−1^; UV(MeOH) λ_max_ (log *ε*) 317 (3.39), 276 (3.77), 236 (3.47), 201 (3.53) nm; HRESIMS *m*/*z* 496.2306 [M + Na]^+^ (calcd for 496.2311, C_26_H_35_NO_7_Na); ^1^H NMR (CD_3_OD, 500 MHz) and ^13^C NMR (CD_3_OD, 125 MHz) see Table 1.

### 3.8. Synthesis of Compound **6**

Then, 4-Hydroxy-6-methyl-2-pyrone (10 mg, 0.079 mmol), glycine (10 mg, 0.133 mmol), and triethyl orthoformate (12 mg, 0.081 mmol) were dissolved in 2-propanol (1 mL). The reaction mixture was stirred overnight at 70 °C. After the reaction, the mixture was distilled under reduced pressure and was subjected to chromatography to afford a pure product **6**.

(*E*)-((6-methyl-2,4-dioxo-2H-pyran-3(4H)-ylidene)methyl)glycine (**6**): LR-MS *m*/*z*: 212.13 [M + H]^+^; ^1^H NMR (600 MHz, CD_3_OD) *δ*_H_ ppm: 8.44 (1H, s, H-3, minor), 8.33 (1H, s, H-3, major), 5.75 (1H, s, H-6), 4.36 (2H, s, H-2, minor), 4.34 (2H, s, H-2, major), 2.17 (3H, s, H-9, minor), 2.16 (3H, s, H-9, major).

### 3.9. Calculation of ECD Spectra

Conformational searches were performed by CONFLEX version 8.0 program (CONFLEX Corporation, Tokyo, Japan) with a molecular mechanics force field (MMFF) within a window of 3.0 kcal/mol. The conformers were further optimized by the B3LYP/6-311G (d,p). The theoretical calculations of the ECD data were conducted using the time-dependent density functional theory (TD-DFT) method at the B3LYP/6-311G (d,p), which were performed with Gaussian 16 software (Gaussian Inc., Wallingford, CT, USA). The calculated ECD spectra data were averaged based on their Boltzmann populations.

## 4. Conclusions

Two restrciticin derivatives (**1** and **2**), along with three known compounds (**3**–**5**) were isolated from the rice medium cultures of the marine-derived fungus *Penicillium janthinellum* 168CLC-17.1. The structures of the isolated compounds were determined by the analysis of their NMR and mass spectrometric data. The absolute configuration of restricticin B (**1**) was established by NOESY correlations, comparison of specific rotation values with those of restricticin derivatives reported and electronic circular dichroism (ECD). Interestingly, the new restricticin **1** possessed an α-pyrone ring connected to the NH of glycine moiety and showed signal duplication as a 3:2 mixture of isomers. Two isolated restricticins exhibited inhibitory activity on NO production in LPS-stimulated BV-2 microglial cells. Moreover, the restricticins suppressed iNOS and COX-2 expression (both at the protein and mRNA levels), and also inhibited the LPS-induced production of pro-inflammatory cytokines. Additionally, compound **1,** possessing α-pyrone moiety and linked to NH. showed stronger activity than **2** containing the *N*-acetyl group. To the best of our knowledge, this is the first report on the anti-neuroinflammatory activity of restrciticins and compounds **3**–**5**.

## Figures and Tables

**Table 1:** ^1^H and ^13^C NMR data for **1** and **2** at 500 MHz and 125 MHz in CD_3_OD (*δ* in ppm, *J* in Hz).

Position	1			2		
	*δ*_H_ (*J* in Hz)	Type	*δ*_C_	*δ*_H_ (*J* in Hz)	Type	*δ*_C_
1′	3.60 (d, 9.5)	CH	84.9	3.60(d, 9.5)	CH	85.0
2′	5.00 (dd, 9.5, 9.5)	CH	70.4	4.98 (dd, 9.5, 9.5)	CH	67.7
3′	3.52, m	CH	81.0	3.47, m	CH	81.3
4′	2.33, m	CH	32.1	2.30, m	CH	32.4
5′	3.63 (d, 12.0)	CH_2_	70.2	3.64 (d, 12.0)	CH_2_	70.3
	3.78 (d, 12.0)			3.75 (d, 12.0)		
1	1.74. s	CH_3_	10.4	1.78. s	CH_3_	10.5
2		C	132.5		C	132.5
3	5.98 (d, 10.5)	CH	129.5	5.99 (d, 10.5)	CH	129.4
4	6.27 (dd, 14.5, 10.0)	CH	125.3	6.27 (dd, 14.5, 10.5)	CH	125.6
5	6.21 (dd, 14.5, 10.0)	CH	134.4	6.23 (dd, 14.5, 10.5)	CH	134.0
6	6.08 (dd, 15.0, 10.5)	CH	130.6	6.10 (dd, 14.5, 10.5)	CH	130.7
7	5.70, (dt, 15.0, 7.0)	CH	135.3	5.72, (dt, 14.5, 7.0)	CH	135.0
8	2.05 (q, 7.0)	CH_2_	34.5	2.07 (q, 7.0)	CH_2_	34.5
9	1.40, m	CH_2_	22.0	1.44, m	CH_2_	22.1
10	0.90 (t, 7.5)	CH_3_	12.6	0.91 (t, 7.0)	CH_3_	12.6
11	1.06 (d, 7.0)	CH_3_	9.5	1.06 (d, 7.5)	CH_3_	9.6
12	3.33, s	OCH_3_	55.0	3.34, s	OCH_3_	55.4
13		C	167.2		C	169.1
			(167.1) ^b^			
14	4.27 (d, 18.0) 4.36 (d, 18.0) (4.29, 4.38) ^b^	CH_2_	50.0	3.73 (d, 18.0)	CH_2_	40.5
			(49.9) ^b^	3.94 (d, 18.0)		
NH ^a^	11.8, brs					
	(10.1) ^b^					
1′′	8.20, s	CH	162.7		C	172.0
	(8.32) ^b^	CH	(161.5) ^b^			
2′′		C	96.6	1.95, s	CH_3_	20.8
			(96.5) ^b^			
3′′		C	184.1			
			(181.7) ^b^			
4′′	5.74, s	CH	106.9			
			(106.6) ^b^			
5′′		C	165.4			
			(165.3) ^b^			
6′′		C	164.9			
7′′	2.51, s	CH_3_	18.7			

The assignments were aided by COSY, NOESY, HSQC, and HMBC NMR spectra. ^a^ Measured in CDCl_3_^b^ NMR chemical shifts recorded for the minor signals observed for compound **1**.

## Supplementary Materials

The following are available online at https://www.mdpi.com/1660-3397/18/9/465/s1, Figures S1–S10, Table S1: HRESI-MS data, ^1^H NMR, ^13^C NMR, COSY, HSQC, HMBC, NOESY and experimental spectra of **1**, Figure S23, Tables S2–S7: ECDs of **1**, Figures S11–S22: LRMS data, ^1^H NMR, ^13^C NMR and experimental spectra of **2**–**5**.
