# Supplementary material for: Anti-Neuroinflammatory Agent, Restricticin B, from the Marine-Derived Fungus Penicillium janthinellum and Its Inhibitory Activity on the NO Production in BV-2 Microglia Cells
Source: Mar Drugs. 2020 Sep 14;18(9):465. doi: 10.3390/md18090465 (PMC7551942; doi:10.3390/md18090465)
Supplement: Supplementary file 1 [file marinedrugs-18-00465-s001.pdf]

Supplementary data

Anti-neuroinflammatory Agent, Restricticin B from the Marine-Derived Fungus *Penicillium janthinellum* and Its Inhibitory Activity on the NO Production in BV-2 Microglia Cells

Byeoung-Kyu Choi[1], Song-Hee Jo[2], Dong-Kuk Choi[2], Phan Thi Hoai Trinh[3], Hwa-Sun Lee[1], Cao Van Anh[1,4], Tran Thi Thanh Van[3], and Hee Jae Shin[1,4,*]

1 Marine Natural Products Chemistry Laboratory, Korea Institute of Ocean Science and Technology, 385 Haeyang-ro, Yeongdo-gu, Busan 49111, Korea; choibk4404@kiost.ac.kr (B.-K.C.), hwasunlee@kiost.ac.kr (H.-S.L.); caovananh@kiost.ac.kr (C.V.A.)

2 Department of Applied Life Science, Graduate school of Konkuk University, Chungju 27478, Korea; wowsong333@naver.com (S.-H.J.), choidk@kku.ac.kr (D.-K.C.)

3 Department of Marine Biotechnology, Nhatrang Institute of Technology Research and Application, Vietnam Academy of Science and Technology, 02 Hung Vuong, Nha Trang 650000, Vietnam; phanhoaitrinh84@gmail.com (P.T.H.T.); tranthanhvan@nitra.vast.vn (T.T.T.V.)

4 Department of Marine Biotechnology, University of Science and Technology (UST), 217 Gajungro, Yuseong-gu, Daejeon 34113, Korea

[*]Corresponding author. Tel.: +82-51-664-3341; fax: +82-51-664-3340; e-mail: shinhj@kiost.ac.kr

Contents

Figure S1. HRESIMS data of restricticin B (**1**).----1

Figure S2. ^{1}H NMR spectrum of restricticin B (**1**).----2

Figure S3. ^{13}C NMR spectrum of restricticin B (**1**). ----2

Figure S4. COSY spectrum of restricticin B (**1**). ----3

Figure S5. HSQC spectrum of restricticin B (**1**). ----4

Figure S6. HMBC spectrum of restricticin B (**1**).----5

Figure S7. ^{1}H NMR spectrum of restricticin B (**1**) in $CDCl_3$.----6

Figure S8. NOESY spectrum of restricticin B (**1**).----7

Table S1. Comparison of ^{1}H and ^{13}C NMR data for **1a** (major) and **1b** (minor) at 500 MHz and 150 MHz in CD_3OD(δ in ppm, J in Hz)----8

Figure S9. NOESY spectrum of restricticin B (**1**) for EXSY correlations. ----9

Figure S10. Comparison of the ^{1}H NMR data between **1** and **6**.----10

Figure S11. HRESIMS data of N-acetyl restricticin (**2**). ----11

Figure S12. ^{1}H NMR spectrum of N-acetyl restricticin (**2**).----12

Figure S13. ^{13}C NMR spectrum of N-acetyl restricticin (**2**).----12

Figure S14. LRMS data of 3,3”-dihydroxy-6’- desmethyl terphenyllin (**3**).----13

Figure S15. ^{1}H NMR spectrum of 3,3”-dihydroxy-6’- desmethyl terphenyllin (**3**). ----14

Figure S16. ^{13}C NMR spectrum of 3,3”-dihydroxy-6’- desmethyl terphenyllin (**3**). ----14

Figure S17. HRESIMS data of fellutanine B (**4**).----15

Figure S18. ^{1}H NMR spectrum of fellutanine B (**4**). ----16

Figure S19. ^{13}C NMR spectrum of fellutanine B (**4**). ----16

Figure S20. LRMS data of 10,23-dihydro-24,25-dehydro aflavinin (**5**). ----17

Figure S21. ^{1}H NMR spectrum of 10,23-dihydro-24,25-dehydro aflavinin (**5**).----18

Figure S22. ^{13}C NMR spectrum of 10,23-dihydro-24,25-dehydro aflavinin (**5**).----19

Figure S23. DFT optimized conformers and populations of restricticin B (1’S,2’R,3’S,4’S) above 5% population.----20

Table S2. Gibbs free energies and Boltzmann distribution of conformers of compound **1**.----20

Table S3−S7. ECD calculation and energy minimized coordinates of conformer **1−5** for all atoms (Å). ----21

Elemental Composition Report

Single Mass Analysis
Tolerance = 5.0 PPM / DBE: min = -1.5, max = 50.0
Element prediction: Off
Number of isotope peaks used for i-FIT = 3

Monoisotopic Mass, Even Electron Ions
70 formula(e) evaluated with 1 results within limits (all results (up to 1000) for each mass)
Elements Used:
C: 1-30 H: 1-40 N: 1-3 O: 1-10 Na: 1-1

Minimum:				-1.5				
Maximum:		100.0	5.0	50.0				
Mass	Calc. Mass	mDa	PPM	DBE	i-FIT	Norm	Conf(%)	Formula
496.2306	496.2311	-0.5	-1.0	9.5	679.3	n/a	n/a	C26 H35 N O7 Na

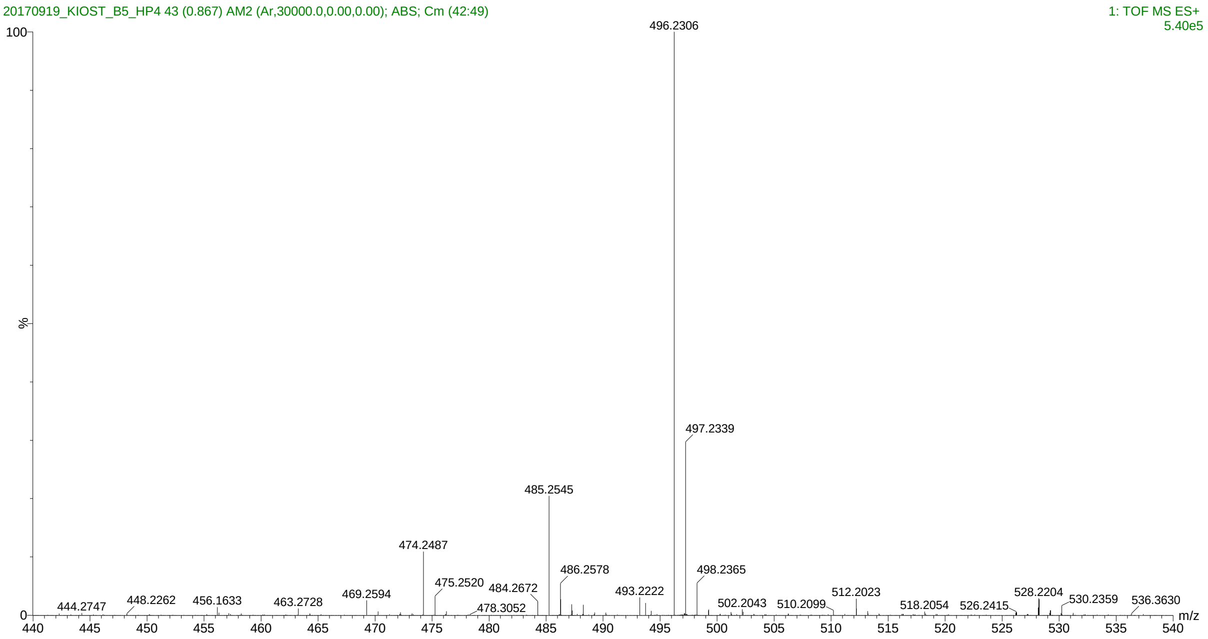

Figure S1. HRESIMS data of restricticin B (**1**).

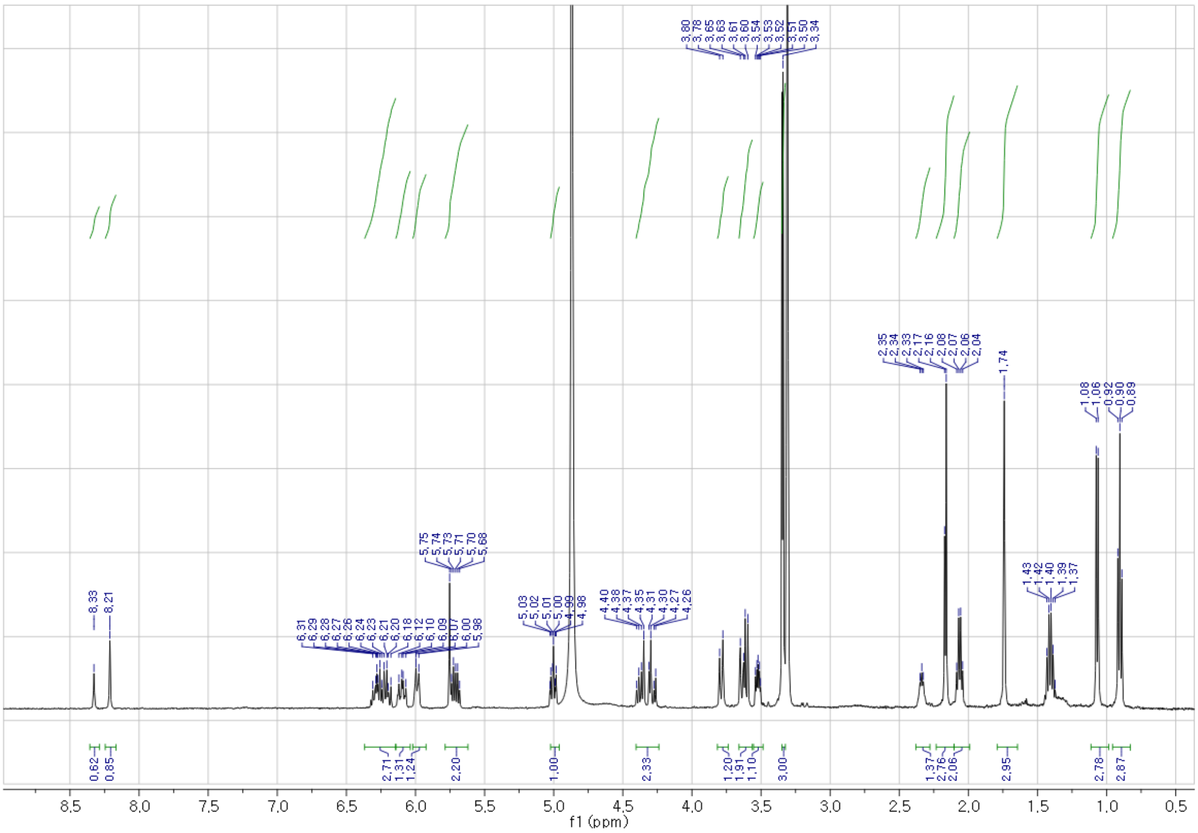

Figure S2. ^{1}H NMR spectrum of restricticin B (**1**).

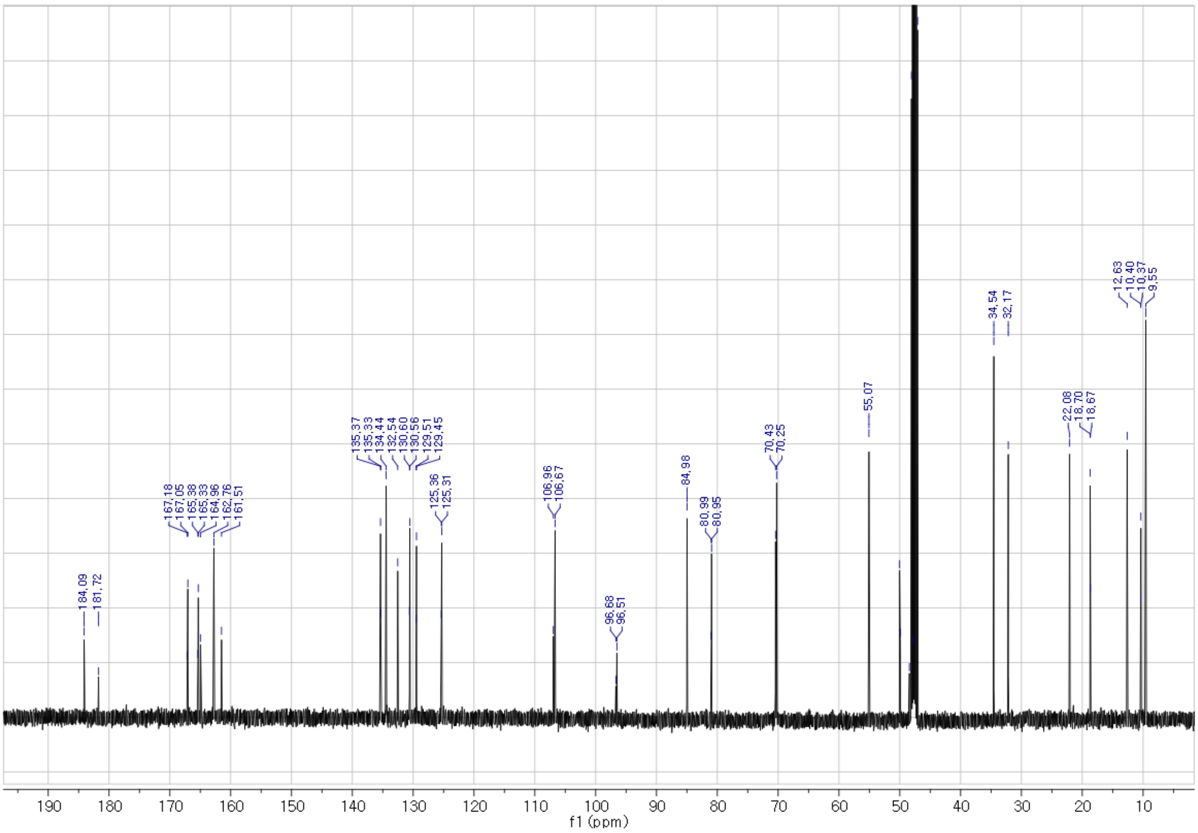

Figure S3. ^{13}C NMR spectrum of restricticin B (**1**).

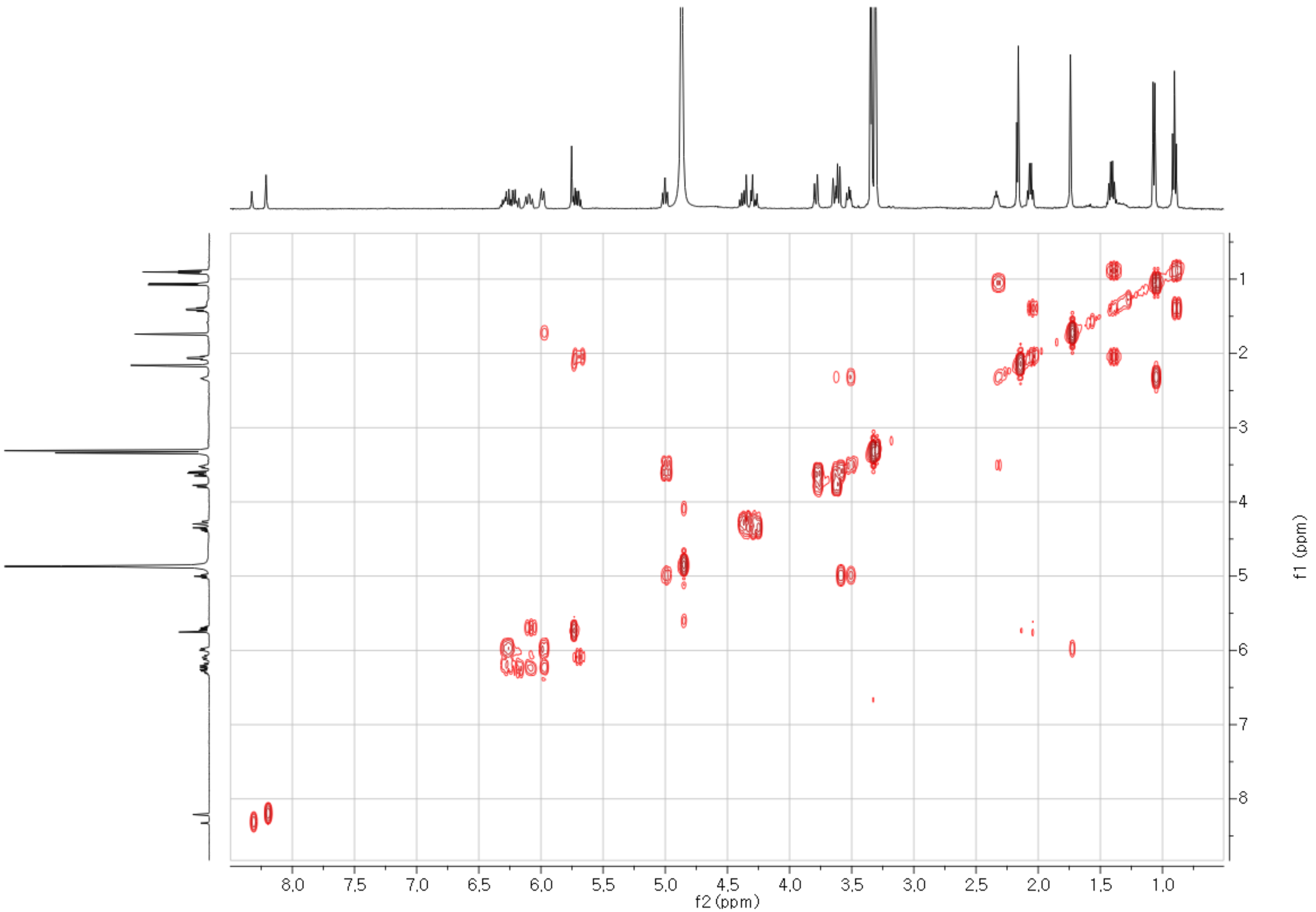

Figure S4. COSY spectrum of restricticin B (**1**).

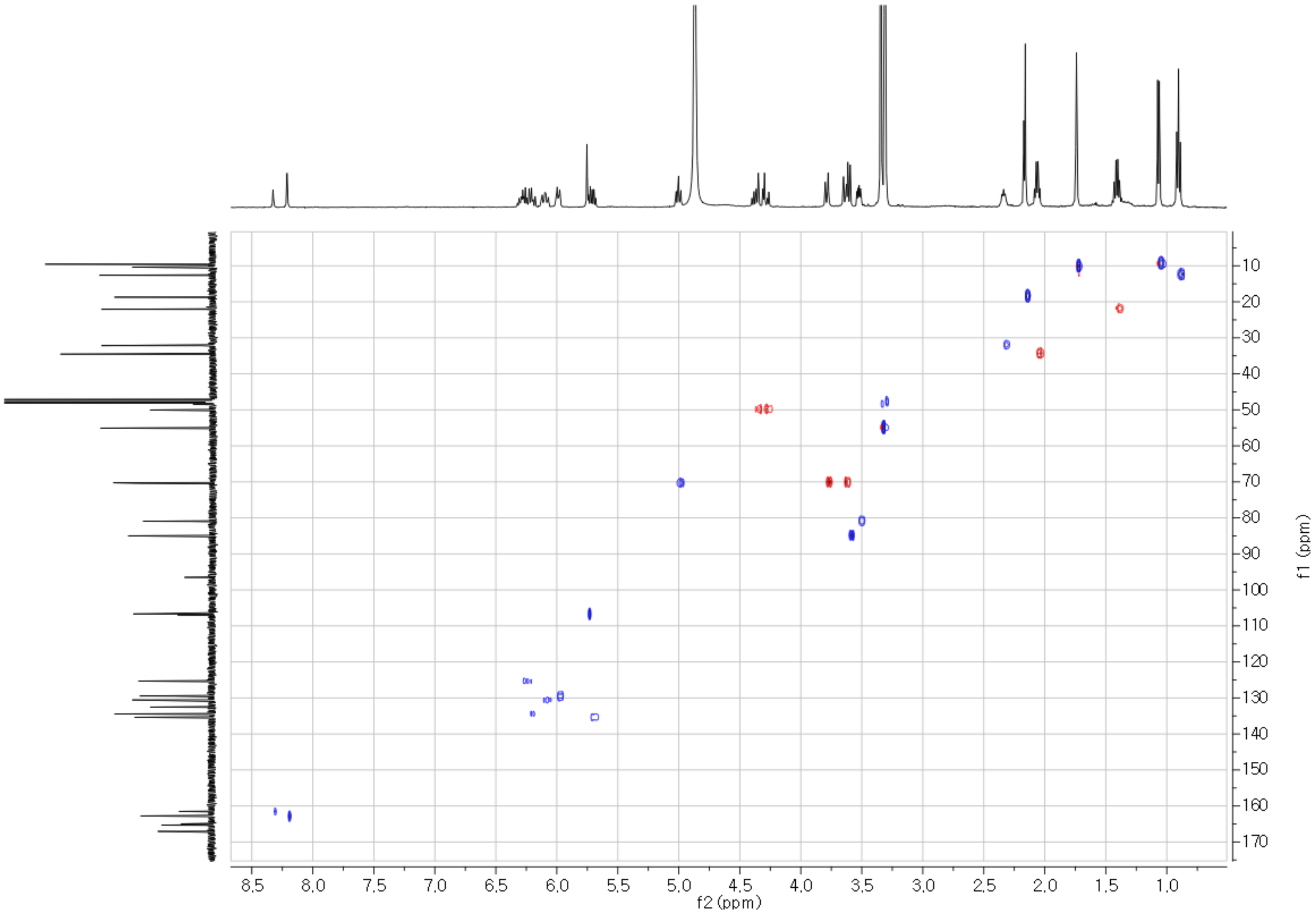

Figure S5. HSQC spectrum of restricticin B (**1**).

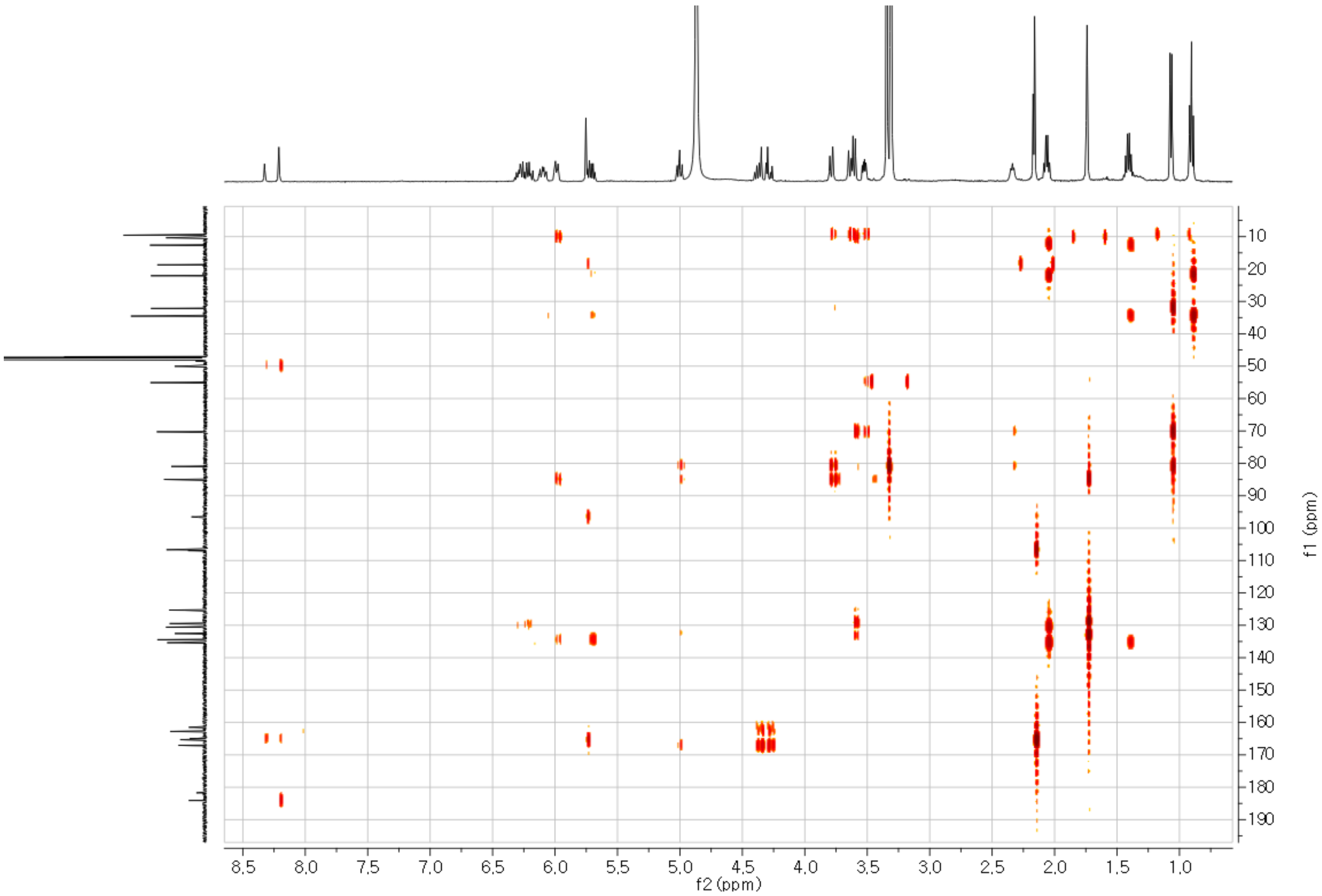

Figure S6. HMBC spectrum of restricticin B (**1**).

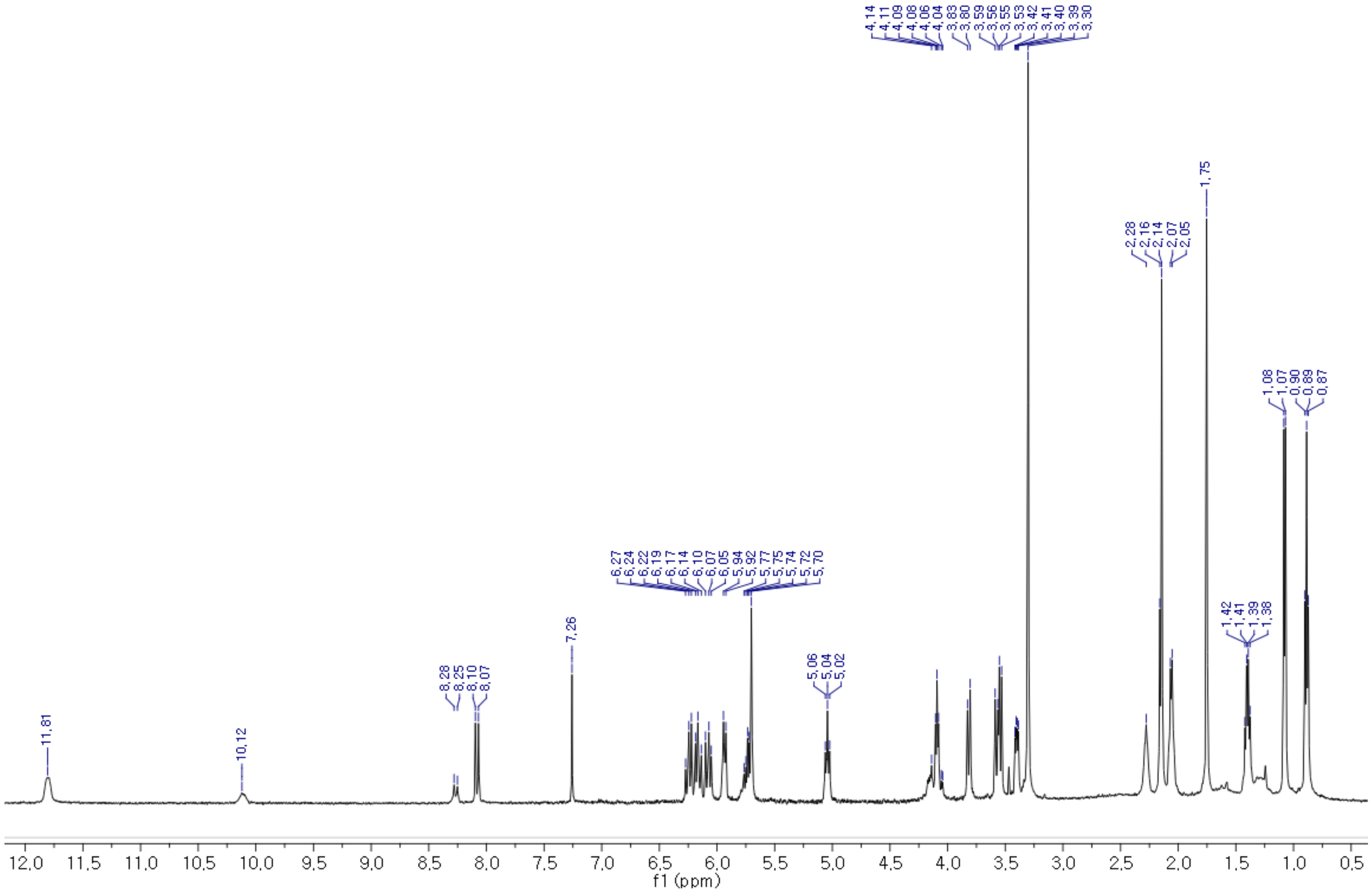

Figure S7. ^{1}H NMR spectrum of restricticin B (**1**) in $CDCl_3$.

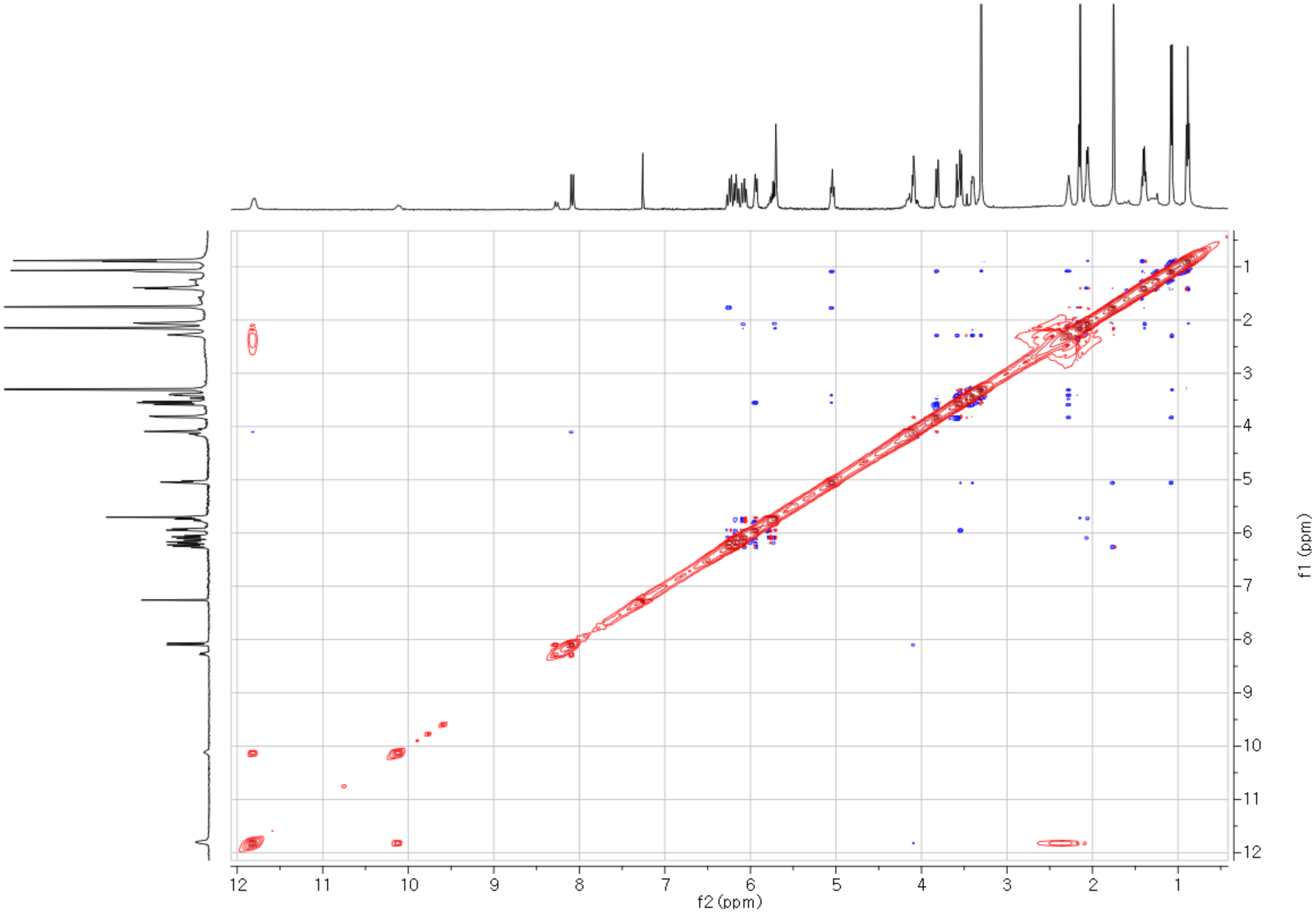

Figure S8. NOESY spectrum of restricticin B (**1**).

Table S1. Comparison of ^{1}H and ^{13}C NMR data for isomer **1** (major) and isomer **1** (minor) at 500 MHz and 150 MHz in CD_3OD(δ in ppm, J in Hz)

Position	**1** (Major)			**1** (Minor)		
	δ_H (J in Hz)	Type	δ_C	δ_H (J in Hz)	Type	δ_C
1′	3.60 (d, 9.5)	CH	84.9	3.60 (d, 9.5)	CH	84.9
2′	5.00 (dd, 9.5, 9.5)	CH	70.4	5.00 (dd, 9.5, 9.5)	CH	70.4
3′	3.52, m	CH	81.0	3.52, m	CH	81.0
4′	2.33, m	CH	32.1	2.33, m	CH	32.1
5′	3.63 (d, 12.0)<br>3.78 (d, 12.0)	CH_2	70.2	3.63 (d, 12.0)<br>3.78 (d, 12.0)	CH_2	70.2
1	1.74. s	CH_3	10.4	1.74. s	CH_3	10.4
2		C	132.5		C	132.5
3	5.98 (d, 10.5)	CH	129.5	5.98 (d, 10.5)	CH	129.5
4	6.27 (dd, 14.5, 10.0)	CH	125.3	6.27 (dd, 14.5, 10.0)	CH	125.3
5	6.21 (dd, 14.5, 10.0)	CH	134.4	6.21 (dd, 14.5, 10.0)	CH	134.4
6	6.08 (dd, 15.0, 10.5)	CH	130.6	6.08 (dd, 15.0, 10.5)	CH	130.6
7	5.70, m	CH	135.3	5.70, m	CH	135.3
8	2.05 (q, 7.0)	CH_2	34.5	2.05 (q, 7.0)	CH_2	34.5
9	1.40, m	CH_2	22.0	1.40, m	CH_2	22.0
10	0.90 (t, 7.5)	CH_3	12.6	0.90 (t, 7.5)	CH_3	12.6
11	1.06 (d, 7.0)	CH_3	9.5	1.06 (d, 7.0)	CH_3	9.5
12	3.33, s	OCH_3	55.0	3.33, s	OCH_3	55.0
13		C	167.2		C	167.1
14	4.27 (d, 18.0)<br>4.36 (d, 18.0)	CH_2	50.0	4.29 (d, 18.0)<br>4.38 (d, 18.0)	CH_2	49.9
NH	11.8, brs			10.1, brs		
1″	8.20, s	CH	162.7	8.32, s	C	161.5
2″		C	96.6		CH_3	96.5
3″		C	184.1			181.7
4″	5.74, s	CH	106.9	5.74, s		106.6
5″		C	165.4			165.3
6″		C	164.9			164.9
7″	2.51, s	CH_3	18.7	2.51, s		18.7

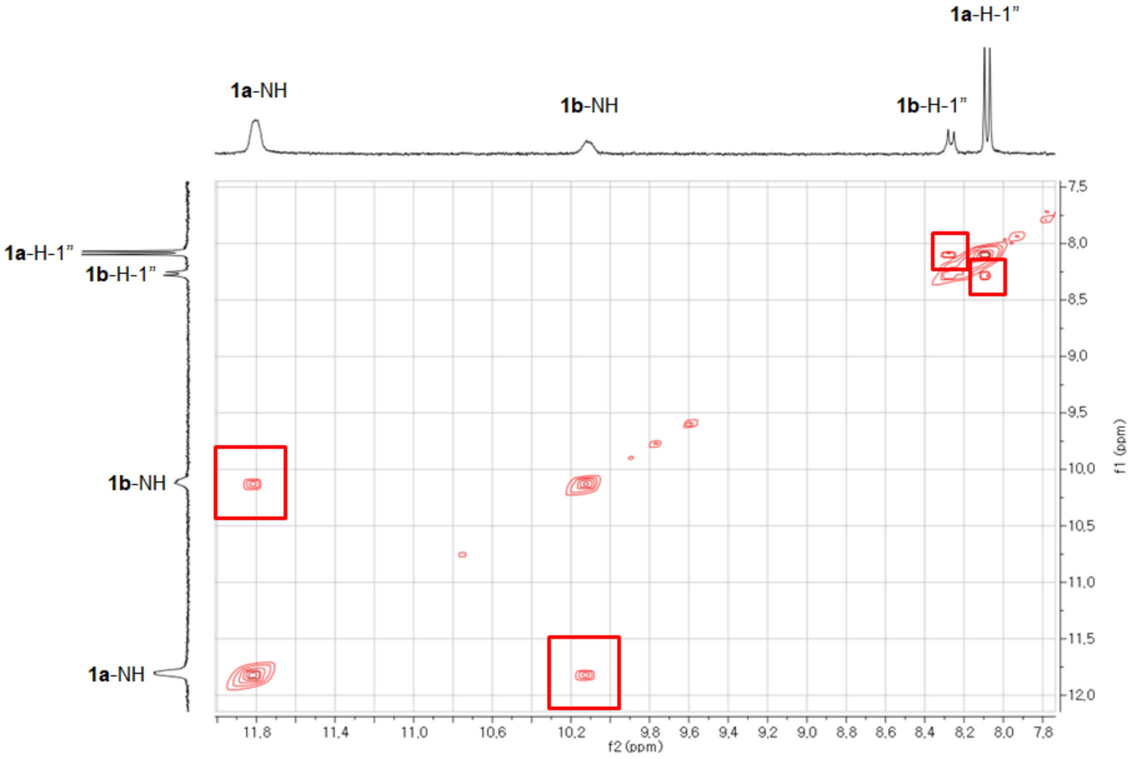

Figure S9. NOESY spectrum of restricticin B (**1**) for EXSY correlations.

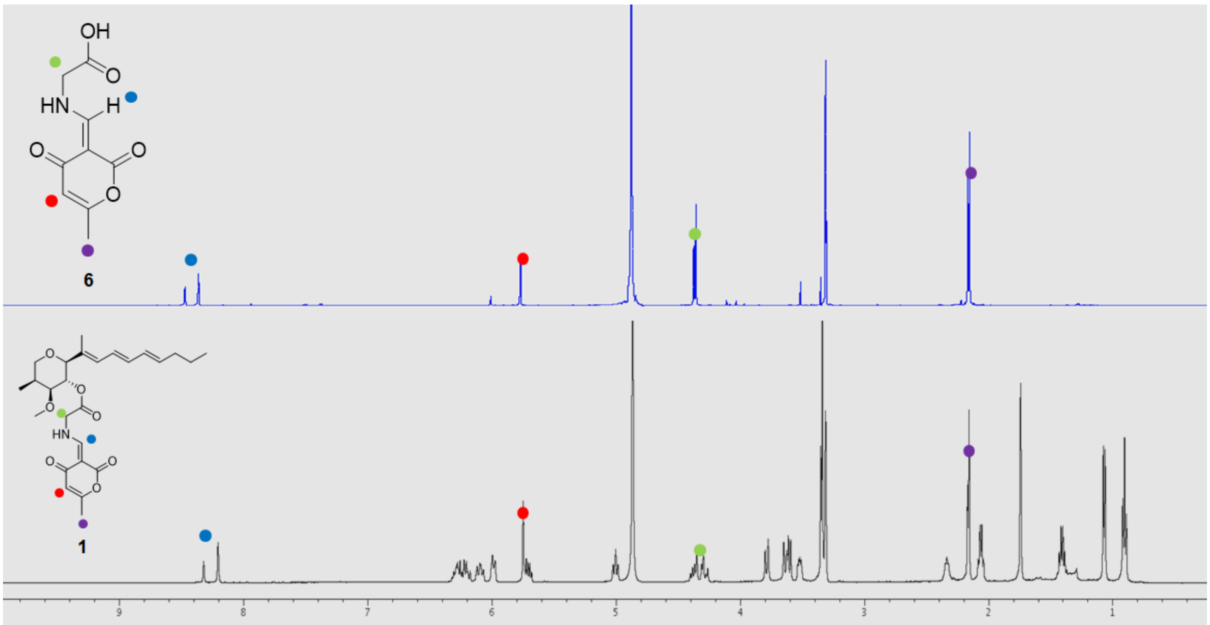

Figure S10. Comparison of the ^{1}H NMR data between **1** and **6**.

Elemental Composition Report

Single Mass Analysis
Tolerance = 5.0 PPM / DBE: min = -1.5, max = 50.0
Element prediction: Off
Number of isotope peaks used for i-FIT = 3

Monoisotopic Mass, Even Electron Ions
118 formula(e) evaluated with 1 results within limits (all results (up to 1000) for each mass)
Elements Used:
C: 1-30 H: 1-50 N: 1-3 O: 1-10 Na: 1-1

Minimum:				-1.5				
Maximum:		100.0	5.0	50.0				
Mass	Calc. Mass	mDa	PPM	DBE	i-FIT	Norm	Conf(%)	Formula
402.2251	402.2256	-0.5	-1.2	5.5	884.7	n/a	n/a	C21 H33 N O5 Na

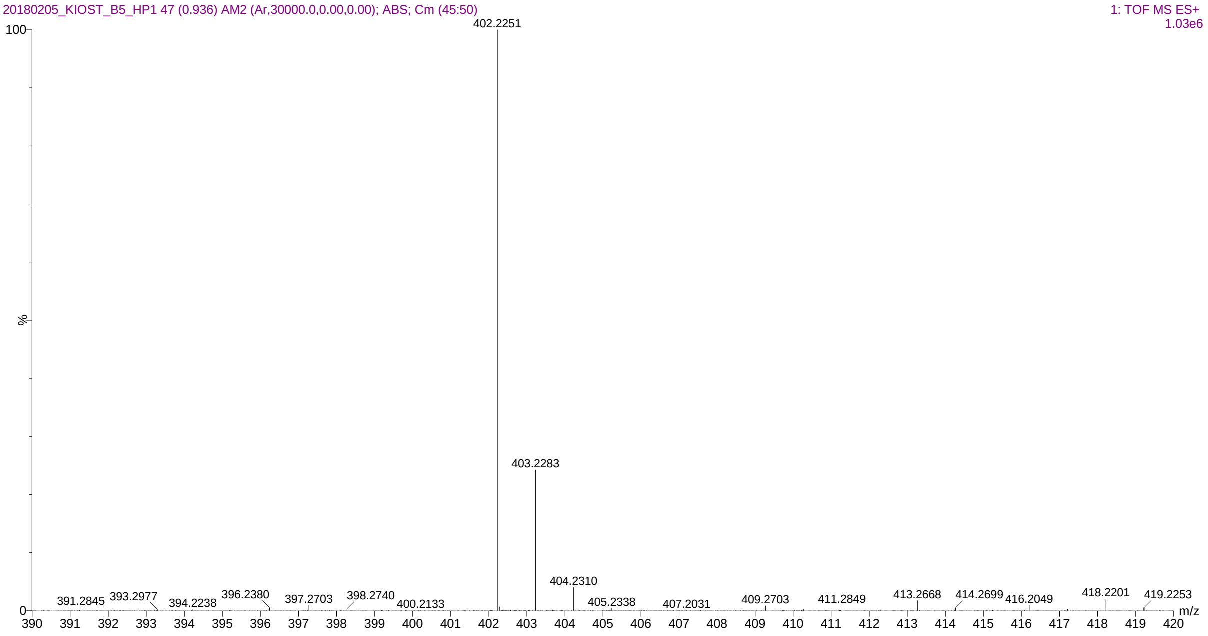

Figure S11. HRESIMS data of N-acetyl restricticin (**2**).

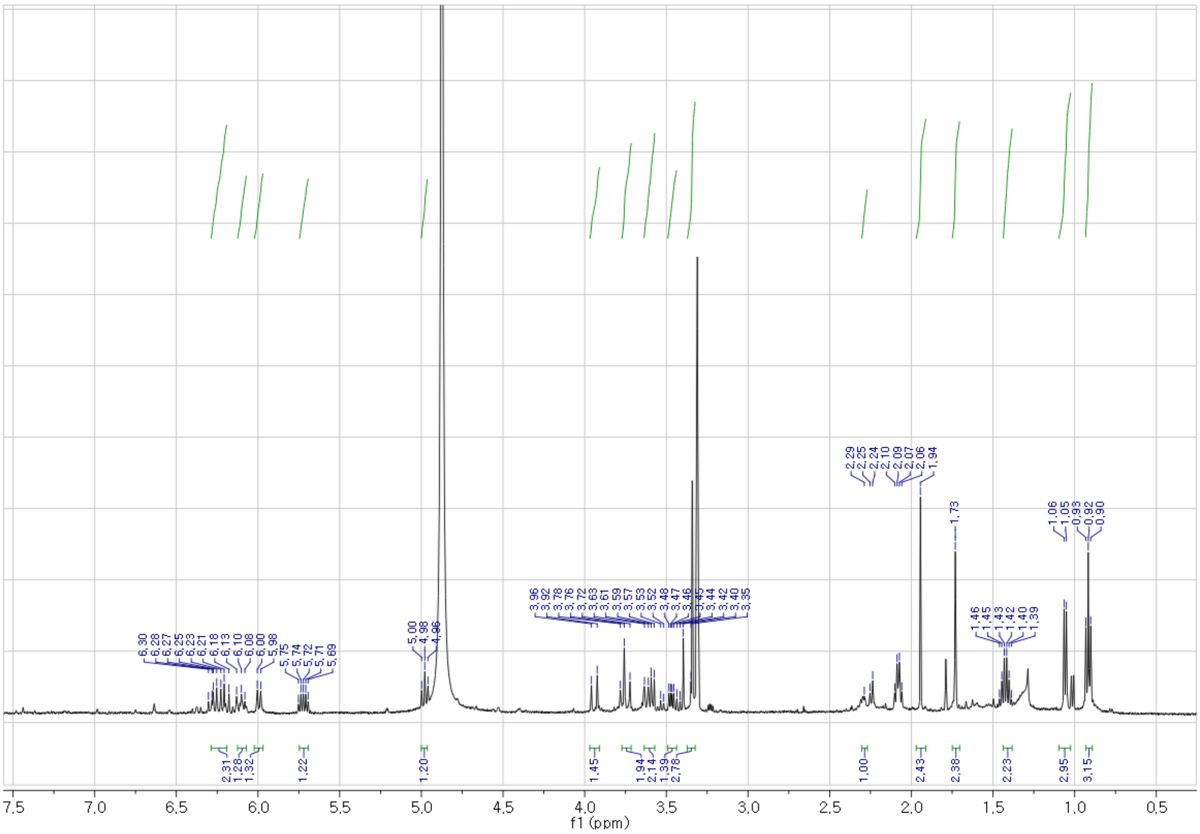

Figure S12. ^{1}H NMR spectrum of N-acetyl restricticin (**2**).

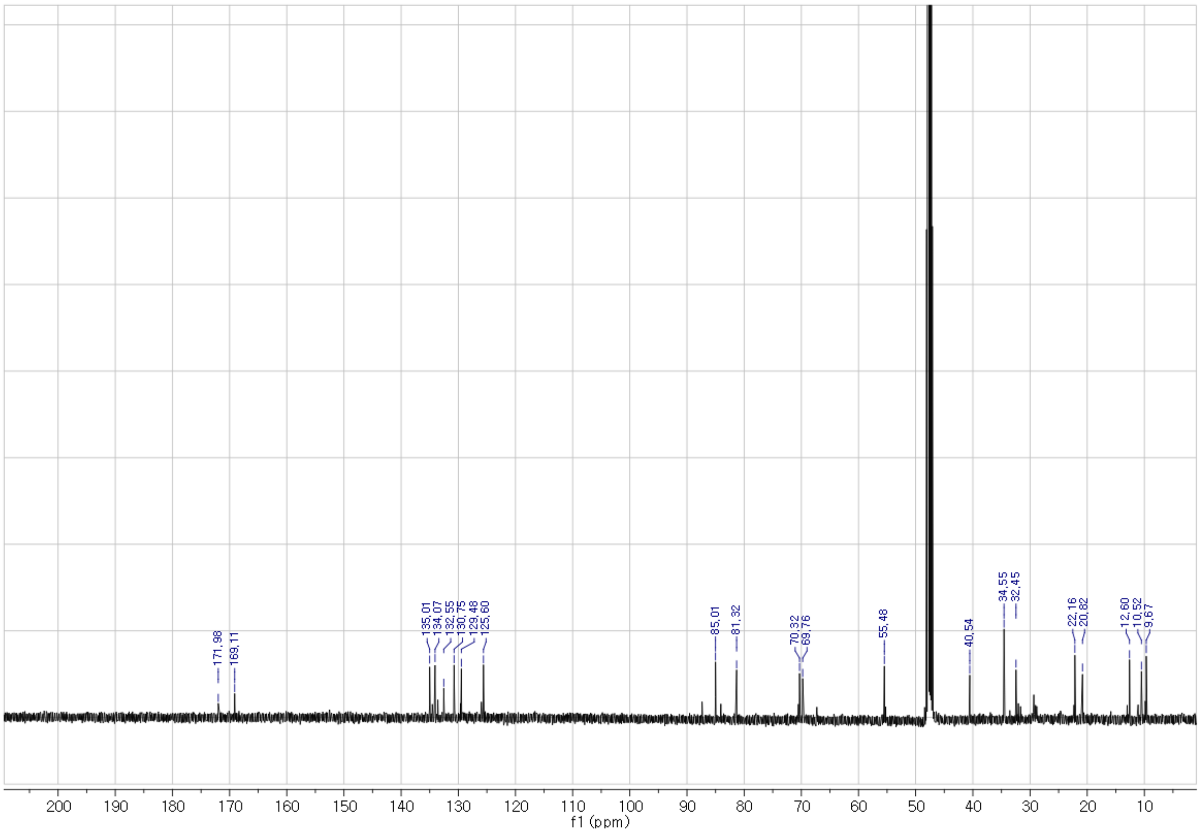

Figure S13. ^{13}C NMR spectrum of N-acetyl restricticin (**2**).

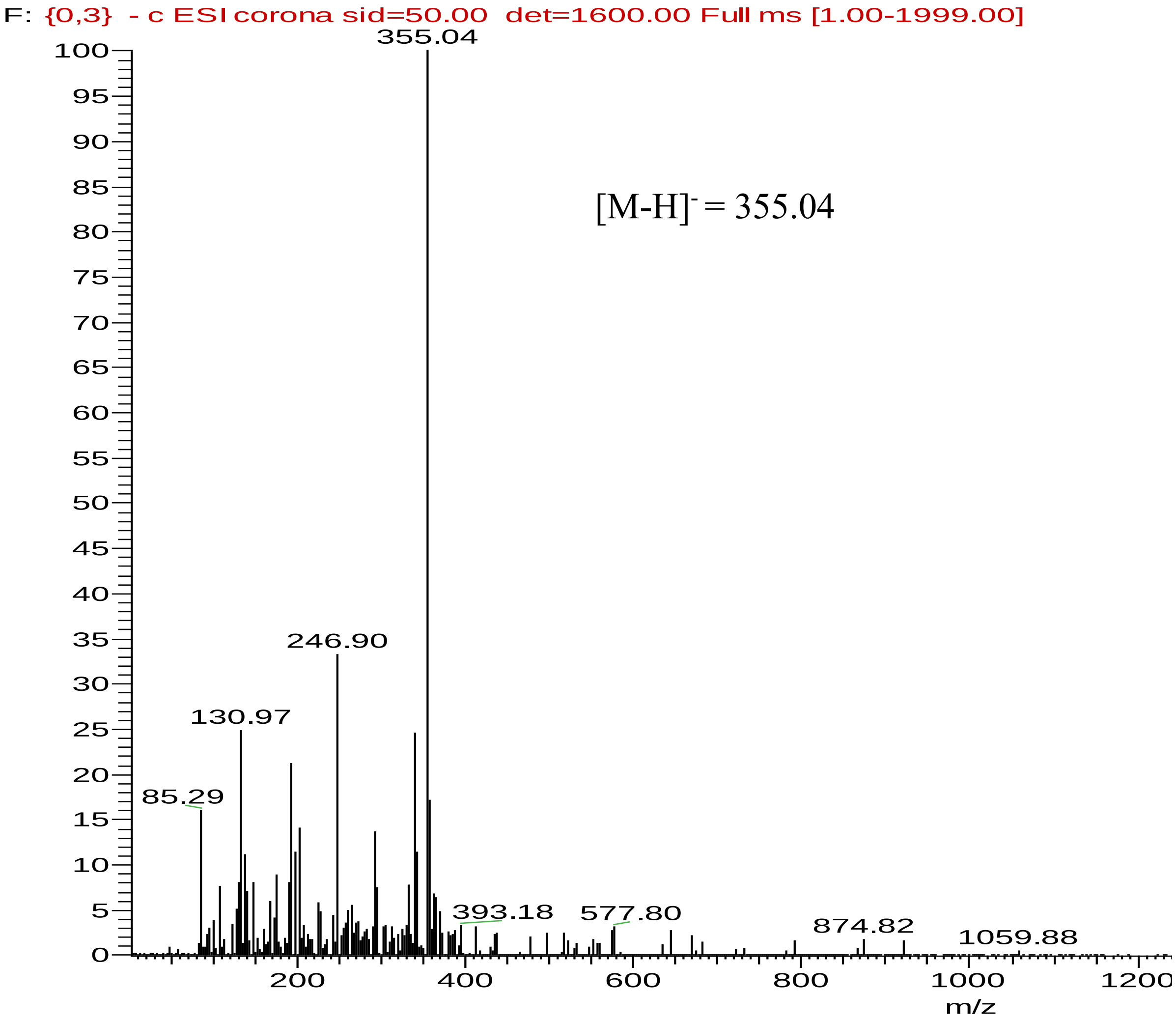

Figure S14. HRESIMS data of 3,3''-dihydroxy-6'- desmethyl terphenyllin (**3**).

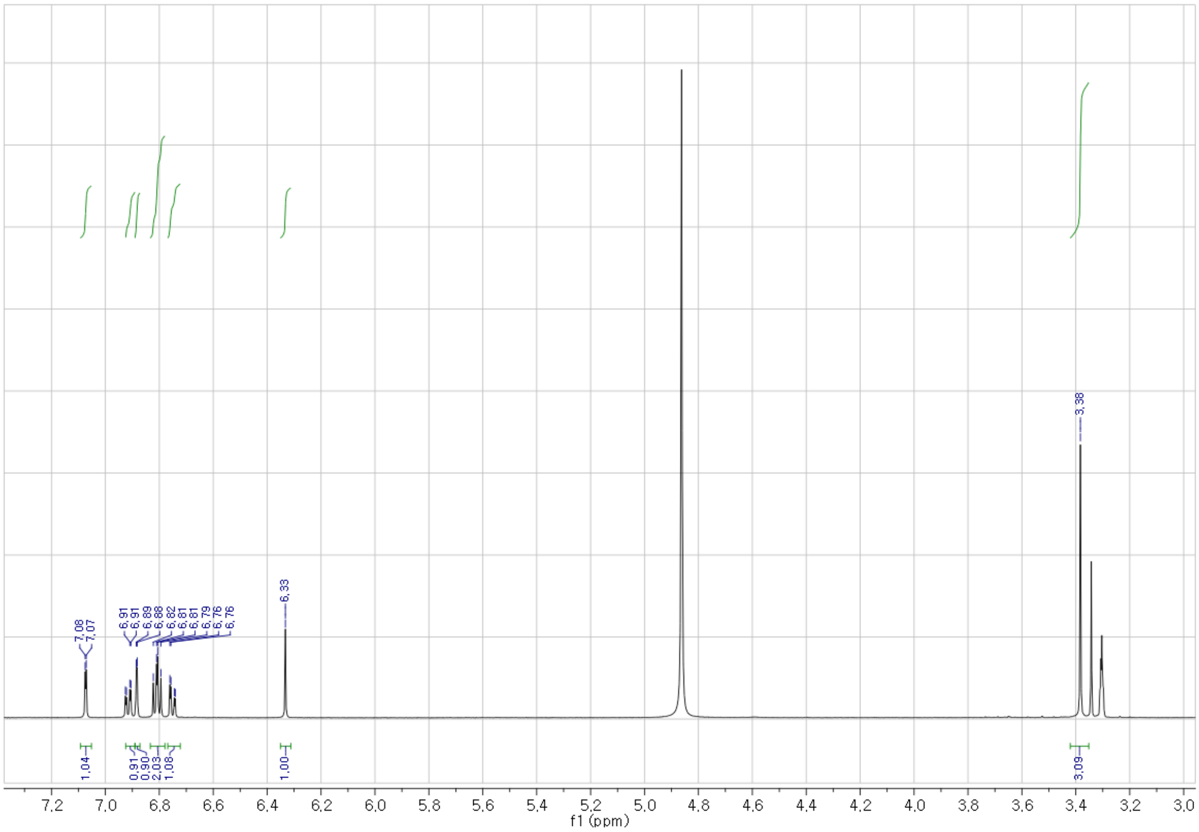

Figure S15. ^{1}H NMR spectrum of 3,3’’-dihydroxy-6’- desmethyl terphenyllin (**3**).

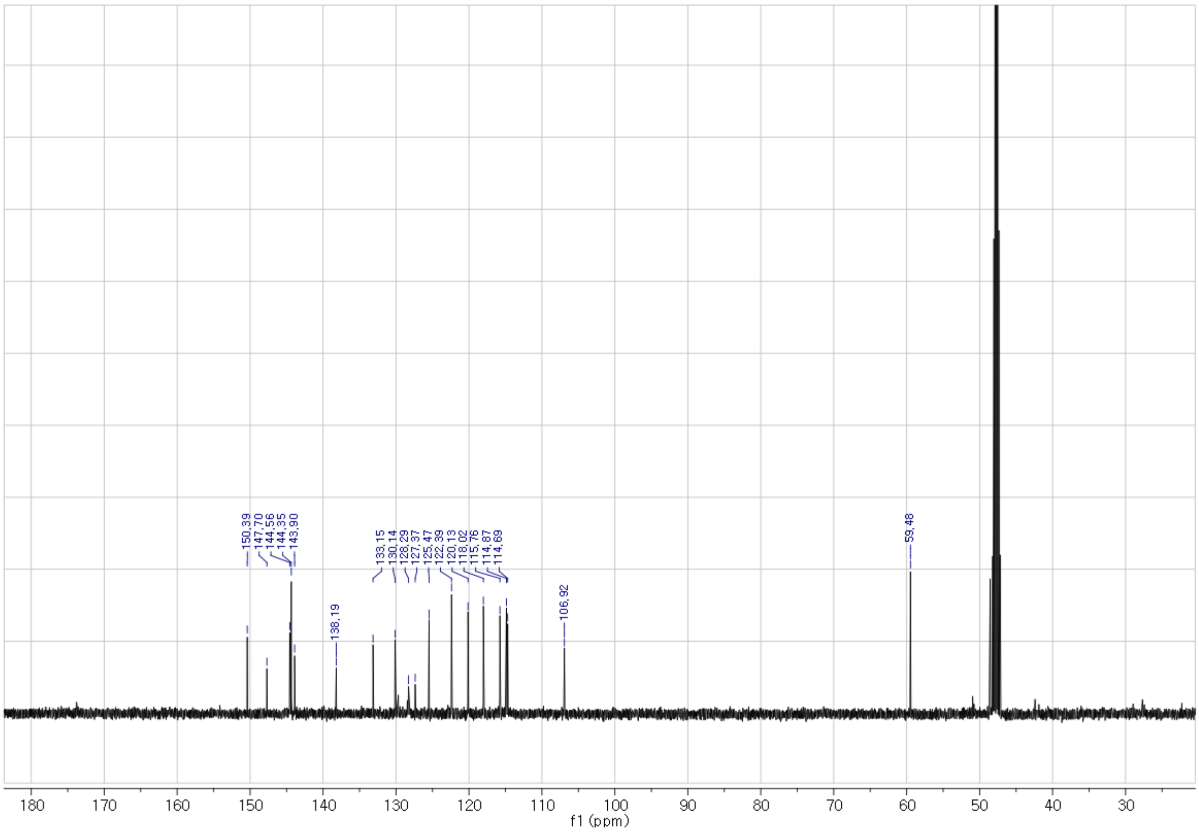

Figure S16. ^{13}C NMR spectrum of 3,3’’-dihydroxy-6’- desmethyl terphenyllin (**3**).

Elemental Composition Report

Single Mass Analysis
Tolerance = 5.0 PPM / DBE: min = -1.5, max = 50.0
Element prediction: Off
Number of isotope peaks used for i-FIT = 3

Monoisotopic Mass, Even Electron Ions
155 formula(e) evaluated with 1 results within limits (all results (up to 1000) for each mass)
Elements Used:
C: 1-30 H: 1-40 N: 1-5 O: 1-10 Na: 1-1

Minimum:				-1.5				
Maximum:		100.0	5.0	50.0				
Mass	Calc. Mass	mDa	PPM	DBE	i-FIT	Norm	Conf(%)	Formula
463.2110	463.2110	0.0	0.0	15.5	720.6	n/a	n/a	C27 H28 N4 O2 Na

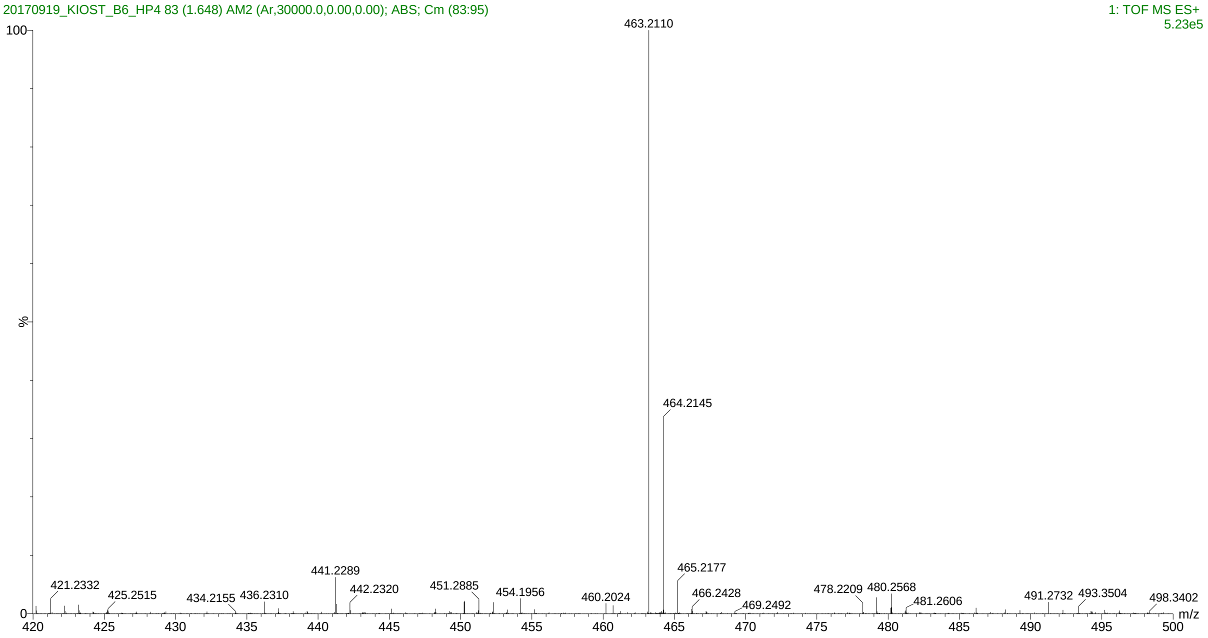

Figure S17. HRESIMS data of fellutanine B (**4**).

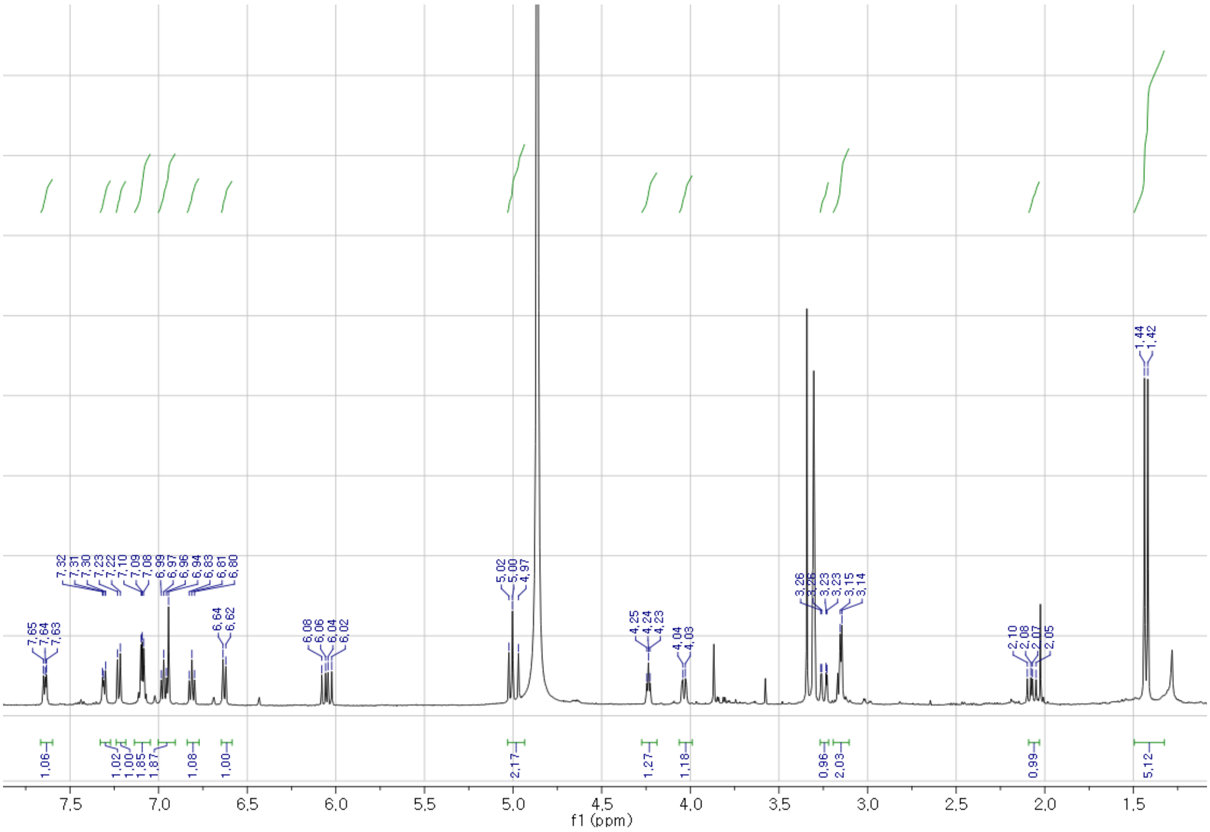

Figure S18. ^{1}H NMR spectrum of fellutanine B (**4**).

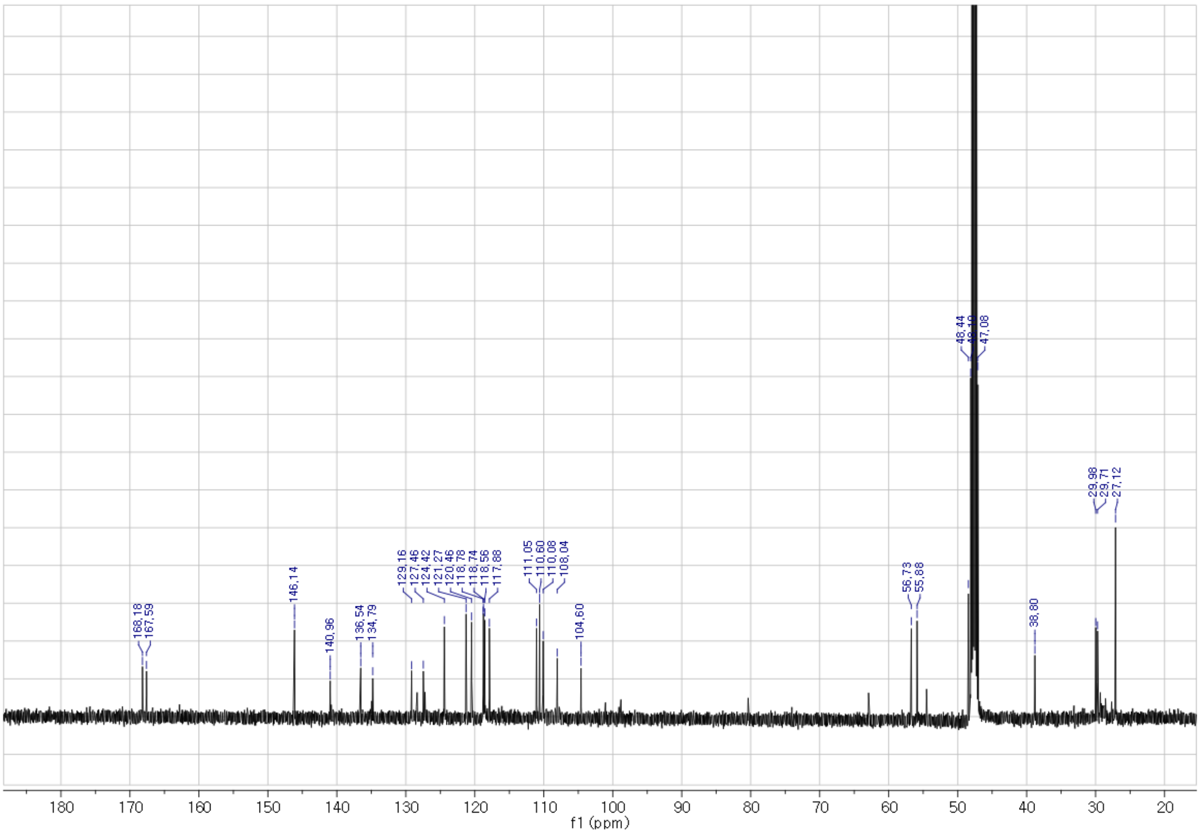

Figure S19. ^{13}C NMR spectrum of fellutanine B (**4**).

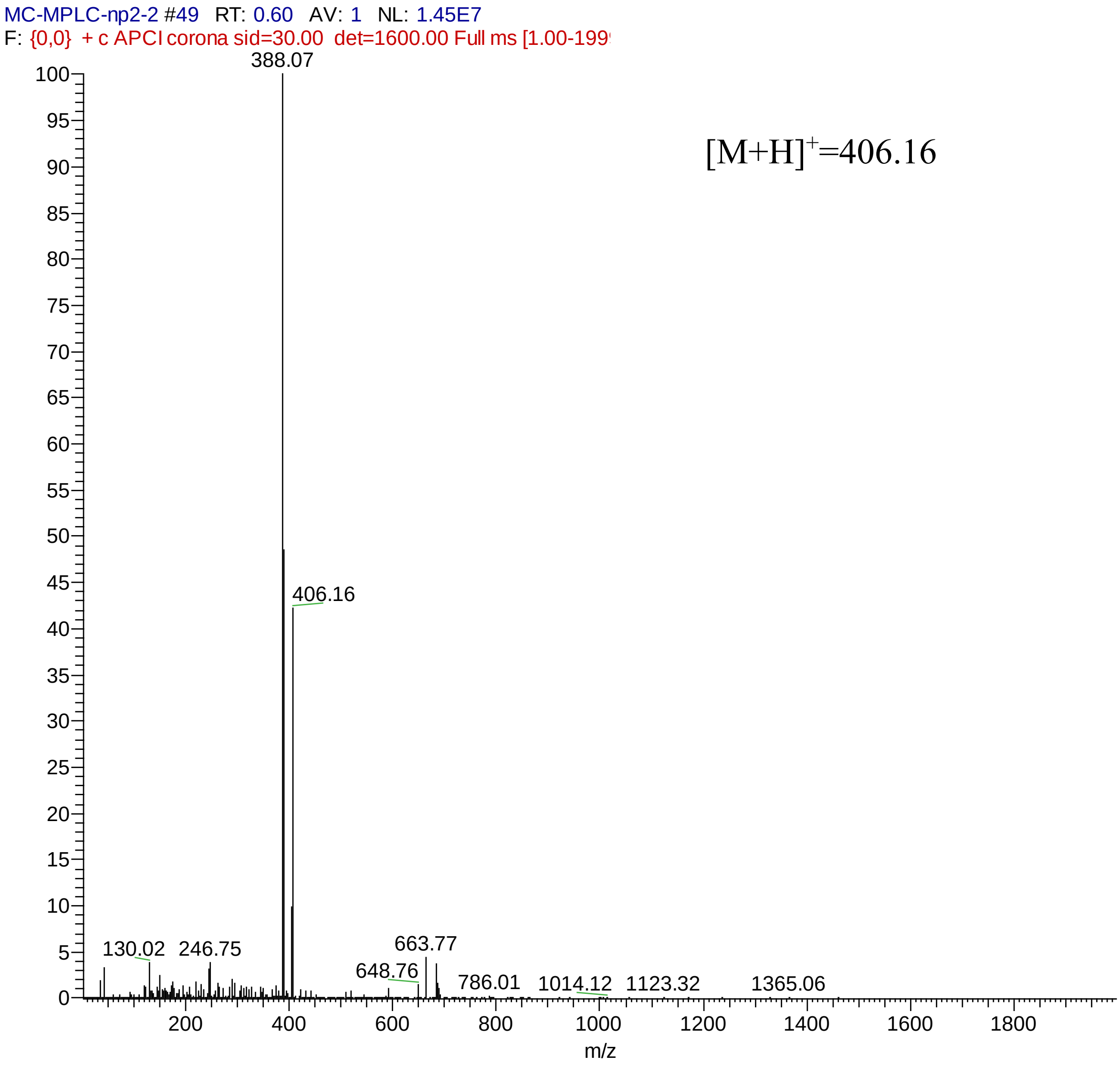

Figure S20. LRMS data of 10,23-dihydro-24,25-dehydro aflavinin (**5**).

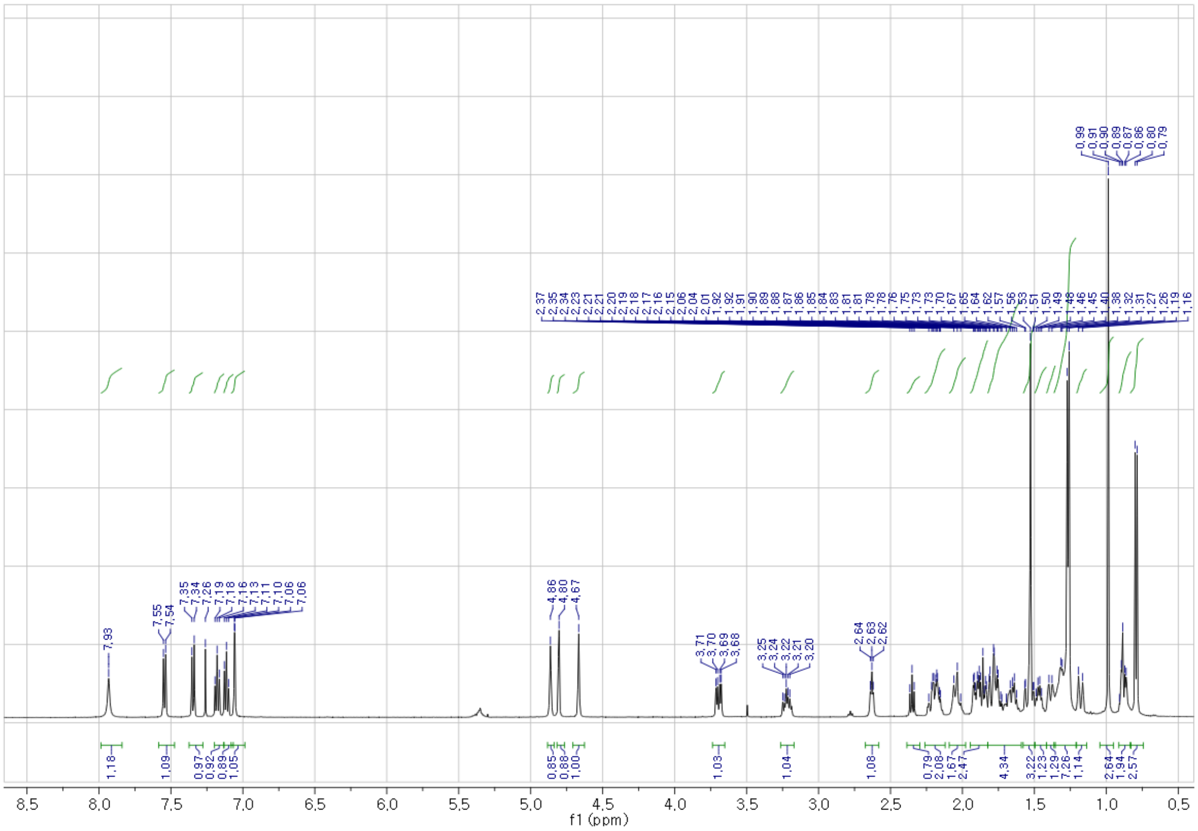

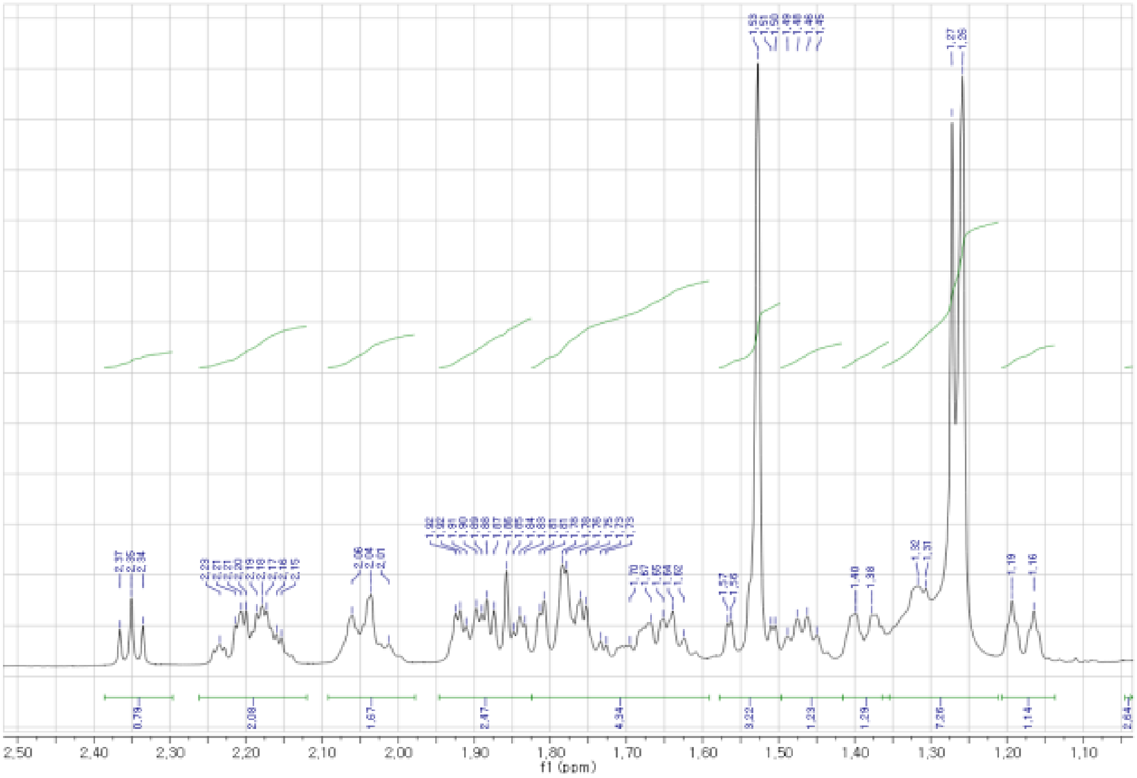

Figure S21. ^{1}H NMR spectrum of 10,23-dihydro-24,25-dehydro aflavinin (**5**).

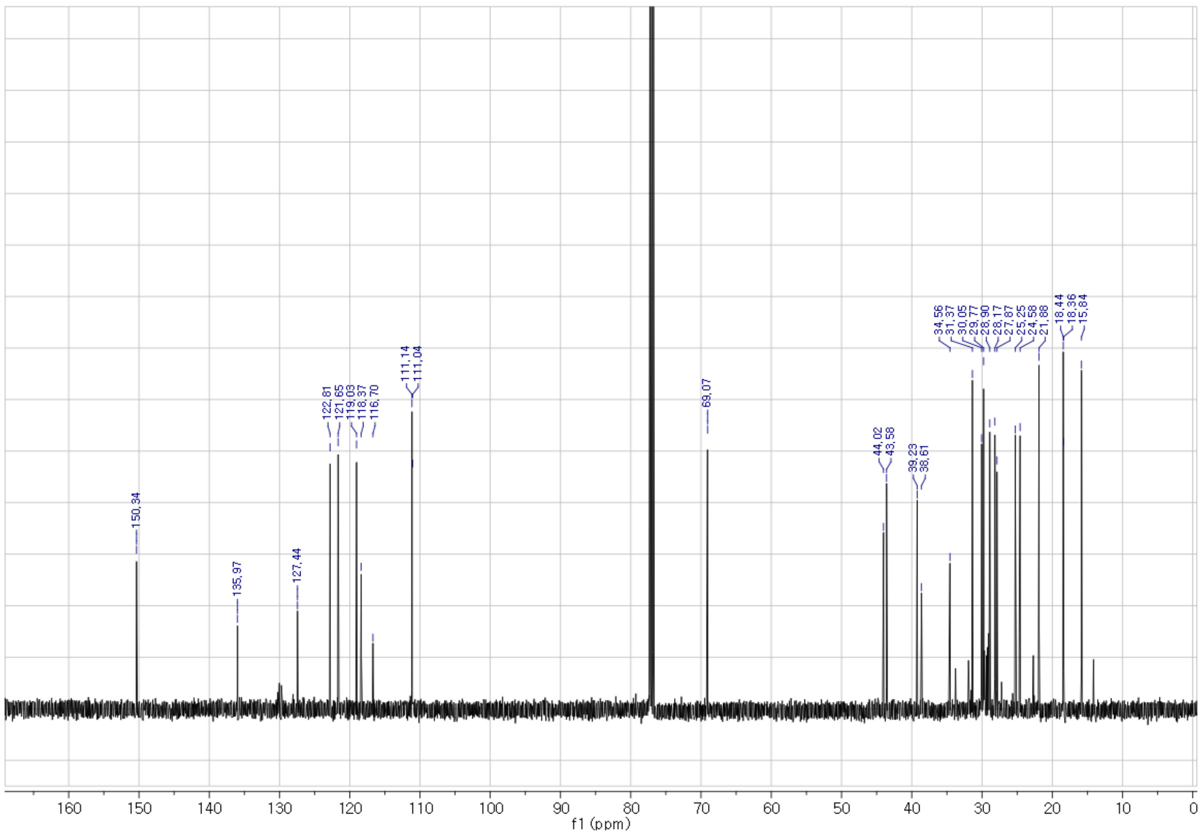

Figure S22. ^{13}C NMR spectrum of 10,23-dihydro-24,25-dehydro aflavinin (**5**).

Figure S23. DFT optimized conformers and populations of restricticin B (1'S,2'R,3'S,4'S) above 5% population.

Conformer **1**

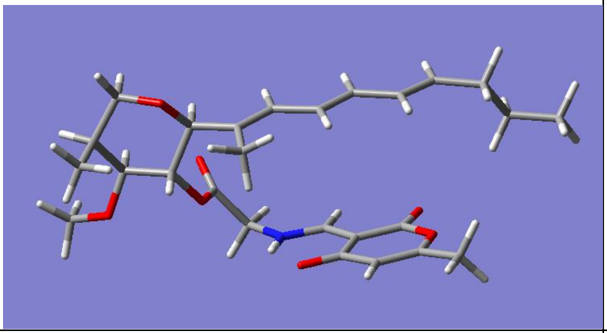

Conformer **2**

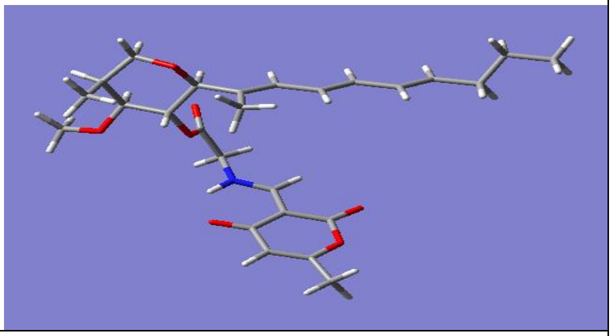

Conformer **3**

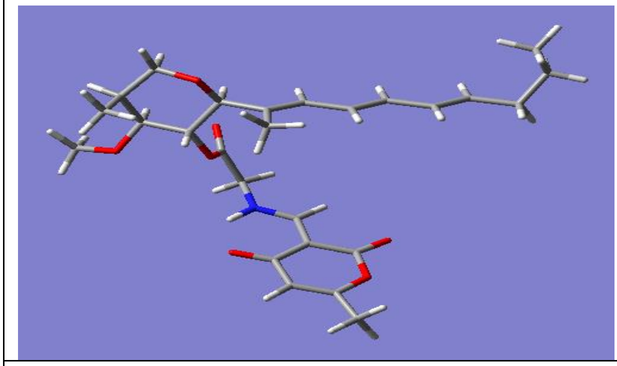

Conformer **4**

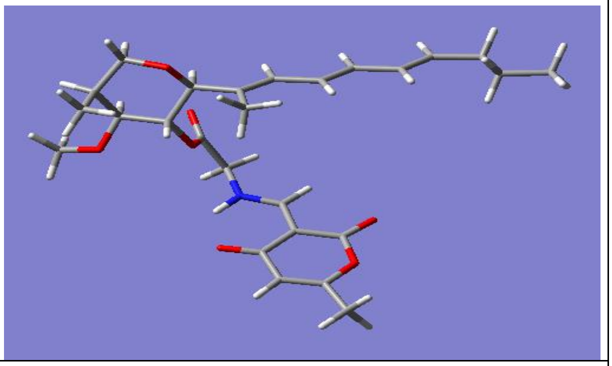

Conformer **5**

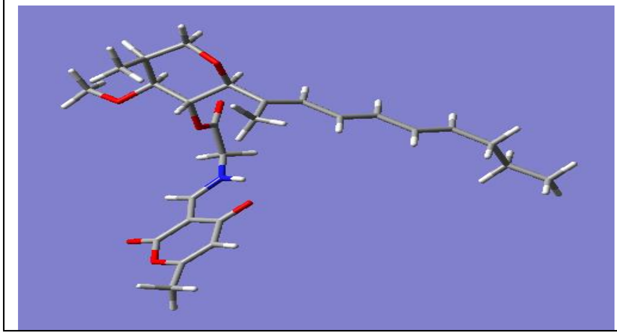

Table S2. Gibbs free energies and Boltzmann distribution of conformers of compound **1**.

B3LYP/6-31+G(d,p) Gibbs free energy (298.15K)		
	G (Hartree)	Population (%)
Conformer 1	-1593.089167	9.48
Conformer 2	-1593.082241	7.93
Conformer 3	-1593.081688	6.00
Conformer 4	-1593.081518	5.64
Conformer 5	-1593.081505	5.54

Table S3. ECD calculation and energy minimized coordinates of conformer **1** for all atoms (Å).

Atom	X	Y	Z	Atom	X	Y	Z
C	-5.8352	0.9565	0.4930	H	-5.6931	2.8867	1.4903
C	-5.1268	2.2903	0.7698	H	-2.9002	1.9911	-0.5002
O	-3.8328	2.0952	1.3488	H	-3.5733	-0.5204	1.1147
C	-2.9365	1.4347	0.4456	H	-4.8600	0.6055	-1.4094
C	-3.5196	0.0248	0.1711	H	-0.7436	2.4215	-0.5780
C	-4.9243	0.1067	-0.4333	H	1.1416	1.4315	1.6912
C	-1.5558	1.3891	1.0562	H	-0.5927	1.0671	2.9738
C	-0.5237	1.9351	0.3725	H	-2.3332	0.7673	2.9641
C	0.8733	1.9098	0.7513	H	-1.2249	-0.4006	2.2271
C	-1.4165	0.6704	2.3762	H	1.6119	2.8835	-0.9700
C	1.8679	2.4125	-0.0201	H	3.5272	1.8736	1.2686
C	3.2747	2.3399	0.3152	H	4.0108	3.2433	-1.4325
C	4.2698	2.7847	-0.4765	H	6.1983	3.6599	-0.1883
C	5.7336	2.6624	-0.1737	H	5.8719	2.2656	0.8401
O	-5.3796	-1.2280	-0.6154	H	-6.6272	-0.7606	1.5772
C	-6.2300	0.2349	1.7889	H	-5.3776	0.1241	2.4653
O	-2.6023	-0.8008	-0.6013	H	-6.9990	0.8063	2.3181
C	-2.2476	-0.4893	-1.8531	H	-6.6693	-2.4146	-1.6357
O	-2.6802	0.4261	-2.5209	H	-7.4005	-0.8912	-1.0803
C	-6.4910	-1.3465	-1.4935	H	-6.2811	-0.8858	-2.4691
C	-1.2172	-1.4953	-2.3704	H	-1.7573	-2.3919	-2.6972
C	6.4791	1.7652	-1.1843	H	-0.7183	-1.0606	-3.2368
C	7.9770	1.6560	-0.8863	H	6.0195	0.7696	-1.1811
N	-0.2385	-1.8505	-1.3589	H	6.3331	2.1673	-2.1955
C	1.0546	-1.5992	-1.4412	H	8.4818	1.0164	-1.6176
C	1.9658	-1.8711	-0.4155	H	8.4594	2.6398	-0.9137
C	1.5317	-2.4181	0.8614	H	8.1531	1.2288	0.1075
C	2.5785	-2.6114	1.8500	H	-0.5378	-2.2495	-0.4598
C	3.8620	-2.2934	1.5770	H	1.4233	-1.1544	-2.3622
O	4.2516	-1.7788	0.3717	H	2.3119	-3.0161	2.8183
C	3.3508	-1.5439	-0.6706	H	5.4998	-1.4717	2.6719
O	3.8261	-1.0828	-1.6945	H	4.6999	-2.8516	3.4637
O	0.3328	-2.7009	1.1015	H	5.7744	-3.1061	2.0667
C	5.0213	-2.4426	2.5052				
H	-6.7495	1.1894	-0.0677				
H	-5.0314	2.8749	-0.1584				

Table S4. ECD calculation and energy minimized coordinates of conformer **2** for all atoms (Å).

Atom	X	Y	Z	Atom	X	Y	Z
C	-4.9736	2.4854	0.6471	H	-4.3849	4.3436	1.6172
C	-3.9882	3.6383	0.8818	H	-1.9230	2.8718	-0.4670
O	-2.7454	3.1705	1.4132	H	-3.0475	0.5503	1.1795
C	-2.0417	2.3357	0.4827	H	-4.1746	1.9399	-1.2892
C	-2.9201	1.0823	0.2355	H	0.2597	2.8422	-0.6247
C	-4.3014	1.4636	-0.3077	H	2.0237	1.6009	1.6174
C	-0.6787	2.0146	1.0530	H	0.3269	1.3782	2.8701
C	0.4182	2.3447	0.3323	H	-1.3790	1.8031	3.0710
C	1.8037	2.1063	0.6792	H	-0.9330	0.2749	2.3146
C	-0.6554	1.3338	2.3975	H	2.6310	2.9783	-1.0550
C	2.8418	2.4763	-0.1098	H	4.4467	1.7475	1.1557
C	4.2359	2.2503	0.2108	H	5.0448	3.1210	-1.5207
C	5.2647	2.6160	-0.5783	H	6.8167	1.8419	0.6731
C	6.7159	2.4017	-0.2653	H	7.1693	1.7795	-1.0521
O	-5.0364	0.2559	-0.4623	H	-6.0592	0.9714	1.7787
C	-5.4559	1.8632	1.9646	H	-4.6175	1.5787	2.6069
O	-2.2463	0.0837	-0.5853	H	-6.0672	2.5823	2.5189
C	-1.9150	0.3328	-1.8542	H	-6.5914	-0.6266	-1.4189
O	-2.0930	1.3705	-2.4590	H	-6.9600	1.0132	-0.8368
C	-6.1861	0.3792	-1.2889	H	-5.9282	0.7913	-2.2746
C	-1.2935	-0.8885	-2.5338	H	-2.0277	-1.2492	-3.2635
C	7.5169	3.7164	-0.1723	H	-0.4204	-0.5482	-3.0941
C	9.0070	3.4852	0.0930	H	7.3912	4.2807	-1.1056
N	-0.9160	-1.9633	-1.6416	H	7.0888	4.3392	0.6231
C	0.3233	-2.3737	-1.4430	H	9.5510	4.4331	0.1563
C	0.6711	-3.4057	-0.5668	H	9.1627	2.9477	1.0353
C	-0.3353	-4.1133	0.2120	H	9.4634	2.8907	-0.7066
C	0.1675	-5.1668	1.0773	H	-1.6187	-2.4557	-1.0758
C	1.4863	-5.4495	1.1369	H	1.1122	-1.8775	-2.0031
O	2.4179	-4.7741	0.3985	H	-0.5373	-5.7265	1.6795
C	2.0755	-3.7381	-0.4762	H	2.8538	-6.0512	2.6617
O	3.0000	-3.2182	-1.0761	H	1.3719	-7.0340	2.5688
O	-1.5563	-3.8370	0.1444	H	2.6603	-7.2193	1.3538
C	2.1222	-6.5022	1.9828				
H	-5.8412	2.9053	0.1221				
H	-3.8068	4.1868	-0.0556				

Table S5. ECD calculation and energy minimized coordinates of conformer **3** for all atoms (Å).

Atom	X	Y	Z	Atom	X	Y	Z
C	-4.8499	2.5517	0.6551	H	-4.2082	4.4127	1.5852
C	-3.8377	3.6878	0.8553	H	-1.8118	2.8569	-0.5138
O	-2.5962	3.2032	1.3744	H	-2.9555	0.5869	1.1903
C	-1.9250	2.3391	0.4466	H	-4.0928	1.9582	-1.2840
C	-2.8323	1.0999	0.2352	H	0.3669	2.7863	-0.7046
C	-4.2140	1.5007	-0.2928	H	2.1442	1.5454	1.5272
C	-0.5591	2.0004	0.9998	H	0.4640	1.3707	2.8094
C	0.5317	2.2998	0.2570	H	-1.2296	1.8325	3.0319
C	1.9183	2.0422	0.5858	H	-0.8265	0.2848	2.2911
C	-0.5267	1.3391	2.3537	H	2.7329	2.8784	-1.1718
C	2.9500	2.3848	-0.2236	H	4.5622	1.6450	1.0263
C	4.3450	2.1406	0.0791	H	5.1401	2.9775	-1.6752
C	5.3674	2.4798	-0.7302	H	6.9306	1.7063	0.5073
C	6.8208	2.2330	-0.4492	H	7.2242	1.5650	-1.2245
O	-4.9760	0.3063	-0.4160	H	-5.9486	1.0799	1.8291
C	-5.3233	1.9611	1.9904	H	-4.4805	1.6690	2.6234
O	-2.1921	0.0736	-0.5783	H	-5.9097	2.7022	2.5427
C	-1.8763	0.2938	-1.8565	H	-6.5634	-0.5594	-1.3339
O	-2.0439	1.3240	-2.4769	H	-6.8894	1.0968	-0.7732
C	-6.1358	0.4398	-1.2267	H	-5.8851	0.8306	-2.2230
C	-1.2894	-0.9512	-2.5237	H	-2.0421	-1.3103	-3.2351
C	7.6763	3.5190	-0.4351	H	-0.4190	-0.6378	-3.1037
C	7.3320	4.4746	0.7106	H	8.7326	3.2312	-0.3687
N	-0.9179	-2.0170	-1.6182	H	7.5593	4.0365	-1.3964
C	0.3158	-2.4511	-1.4359	H	7.9633	5.3686	0.6831
C	0.6582	-3.4744	-0.5474	H	6.2875	4.7988	0.6586
C	-0.3479	-4.1456	0.2634	H	7.4796	3.9914	1.6835
C	0.1489	-5.1940	1.1382	H	-1.6201	-2.4832	-1.0300
C	1.4623	-5.5044	1.1780	H	1.1041	-1.9829	-2.0203
O	2.3935	-4.8630	0.4095	H	-0.5559	-5.7272	1.7640
C	2.0566	-3.8360	-0.4777	H	2.8460	-6.1078	2.6872
O	2.9801	-3.3477	-1.1050	H	1.3417	-7.0595	2.6412
O	-1.5637	-3.8438	0.2140	H	2.6019	-7.2951	1.4053
C	2.0918	-6.5550	2.0311				
H	-5.7172	2.9807	0.1370				
H	-3.6605	4.2175	-0.0938				

Table S6. ECD calculation and energy minimized coordinates of conformer **4** for all atoms (Å).

Atom	X	Y	Z	Atom	X	Y	Z
C	-4.8499	2.5517	0.6551	H	-4.2082	4.4127	1.5852
C	-3.8377	3.6878	0.8553	H	-1.8118	2.8569	-0.5138
O	-2.5962	3.2032	1.3744	H	-2.9555	0.5869	1.1903
C	-1.9250	2.3391	0.4466	H	-4.0928	1.9582	-1.2840
C	-2.8323	1.0999	0.2352	H	0.3669	2.7863	-0.7046
C	-4.2140	1.5007	-0.2928	H	2.1442	1.5454	1.5272
C	-0.5591	2.0004	0.9998	H	0.4640	1.3707	2.8094
C	0.5317	2.2998	0.2570	H	-1.2296	1.8325	3.0319
C	1.9183	2.0422	0.5858	H	-0.8265	0.2848	2.2911
C	-0.5267	1.3391	2.3537	H	2.7329	2.8784	-1.1718
C	2.9500	2.3848	-0.2236	H	4.5622	1.6450	1.0263
C	4.3450	2.1406	0.0791	H	5.1401	2.9775	-1.6752
C	5.3674	2.4798	-0.7302	H	6.9306	1.7063	0.5073
C	6.8208	2.2330	-0.4492	H	7.2242	1.5650	-1.2245
O	-4.9760	0.3063	-0.4160	H	-5.9486	1.0799	1.8291
C	-5.3233	1.9611	1.9904	H	-4.4805	1.6690	2.6234
O	-2.1921	0.0736	-0.5783	H	-5.9097	2.7022	2.5427
C	-1.8763	0.2938	-1.8565	H	-6.5634	-0.5594	-1.3339
O	-2.0439	1.3240	-2.4769	H	-6.8894	1.0968	-0.7732
C	-6.1358	0.4398	-1.2267	H	-5.8851	0.8306	-2.2230
C	-1.2894	-0.9512	-2.5237	H	-2.0421	-1.3103	-3.2351
C	7.6763	3.5190	-0.4351	H	-0.4190	-0.6378	-3.1037
C	7.3320	4.4746	0.7106	H	8.7326	3.2312	-0.3687
N	-0.9179	-2.0170	-1.6182	H	7.5593	4.0365	-1.3964
C	0.3158	-2.4511	-1.4359	H	7.9633	5.3686	0.6831
C	0.6582	-3.4744	-0.5474	H	6.2875	4.7988	0.6586
C	-0.3479	-4.1456	0.2634	H	7.4796	3.9914	1.6835
C	0.1489	-5.1940	1.1382	H	-1.6201	-2.4832	-1.0300
C	1.4623	-5.5044	1.1780	H	1.1041	-1.9829	-2.0203
O	2.3935	-4.8630	0.4095	H	-0.5559	-5.7272	1.7640
C	2.0566	-3.8360	-0.4777	H	2.8460	-6.1078	2.6872
O	2.9801	-3.3477	-1.1050	H	1.3417	-7.0595	2.6412
O	-1.5637	-3.8438	0.2140	H	2.6019	-7.2951	1.4053
C	2.0918	-6.5550	2.0311				
H	-5.7172	2.9807	0.1370				
H	-3.6605	4.2175	-0.0938				

Table S7. ECD calculation and energy minimized coordinates of conformer **5** for all atoms (Å).

Atom	X	Y	Z	Atom	X	Y	Z
C	-3.2052	4.1731	0.5704	H	-2.0326	5.6511	1.6571
C	-1.8802	4.8737	0.9034	H	-0.1844	3.4479	-0.4073
O	-0.9239	3.9658	1.4592	H	-2.1554	1.6684	1.1120
C	-0.5302	2.9608	0.5133	H	-2.5543	3.4174	-1.3532
C	-1.7925	2.1244	0.1896	H	1.8894	2.7620	-0.4254
C	-2.9034	2.9990	-0.3996	H	2.9551	0.7981	1.7398
C	0.5983	2.1463	1.1032	H	1.1904	1.1243	2.9253
C	1.7968	2.1542	0.4748	H	-0.3773	1.9365	3.0078
C	2.9933	1.4340	0.8575	H	-0.2115	0.4174	2.1207
C	0.2907	1.3651	2.3561	H	4.2013	2.1398	-0.7214
C	4.1573	1.5085	0.1671	H	5.3265	0.1687	1.4110
C	5.3699	0.8015	0.5235	H	6.5583	1.5182	-1.0527
C	6.5252	0.8845	-0.1644	H	8.5921	0.8915	0.3641
C	7.7924	0.1584	0.1772	H	7.6604	-0.4051	1.1095
O	-4.0213	2.1533	-0.6380	H	-4.8384	3.1420	1.5815
C	-3.9600	3.7402	1.8349	H	-3.3260	3.1478	2.5009
O	-1.4851	0.9557	-0.6219	H	-4.2933	4.6204	2.3936
C	-1.0027	1.0682	-1.8610	H	-5.7338	1.9307	-1.7011
O	-0.7965	2.0990	-2.4684	H	-5.5061	3.5765	-1.0660
C	-4.9951	2.7120	-1.5096	H	-4.5469	3.0224	-2.4640
C	-0.7226	-0.3013	-2.4870	H	-1.4541	-0.4602	-3.2847
C	8.2656	-0.7931	-0.9406	H	0.2635	-0.2399	-2.9563
C	9.5885	-1.4881	-0.6069	H	7.4862	-1.5432	-1.1239
N	-0.7687	-1.4087	-1.5583	H	8.3720	-0.2257	-1.8744
C	-1.6966	-2.3477	-1.5451	H	9.9003	-2.1576	-1.4150
C	-1.7652	-3.3562	-0.5796	H	10.3903	-0.7577	-0.4499
C	-0.8228	-3.4123	0.5287	H	9.5020	-2.0864	0.3072
C	-1.0251	-4.5002	1.4698	H	-0.1082	-1.4654	-0.7719
C	-2.0274	-5.3893	1.3023	H	-2.4426	-2.3287	-2.3360
O	-2.9030	-5.3223	0.2545	H	-0.3535	-4.5892	2.3145
C	-2.8276	-4.3265	-0.7243	H	-2.2837	-7.4829	1.6306
O	-3.6688	-4.3775	-1.6044	H	-1.6313	-6.5908	3.0267
O	0.1040	-2.5784	0.6688	H	-3.3525	-6.4583	2.5900
C	-2.3348	-6.5456	2.1947				
H	-3.8242	4.8985	0.0271				
H	-1.4591	5.3475	0.0031				
